# Fractional Dynamics Foster Deep Learning of COPD Stage Prediction

**DOI:** 10.1002/advs.202203485

**Published:** 2023-02-19

**Authors:** Chenzhong Yin, Mihai Udrescu, Gaurav Gupta, Mingxi Cheng, Andrei Lihu, Lucretia Udrescu, Paul Bogdan, David M. Mannino, Stefan Mihaicuta

**Affiliations:** ^1^ Ming Hsieh Department of Electrical and Computer Engineering University of Southern California Los Angeles CA USA; ^2^ Department of Computer and Information Technology Politehnica University of Timisoara 2 Vasile Parvan Blvd. Timişoara 300223 Romania; ^3^ Department I – Drug Analysis “Victor Babeş” University of Medicine and Pharmacy Timişoara 2 Eftimie Murgu Sq. Timişoara 300041 Romania; ^4^ College of Medicine University of Kentucky Lexington KY USA; ^5^ Department of Pulmonology Center for Research and Innovation in Precision Medicine of Respiratory Diseases, “Victor Babes” University of Medicine and Pharmacy 2 Eftimie Murgu Sq. Timişoara 300041 Romania

**Keywords:** chronic obstructive pulmonary disease (COPD), deep learning, fractional analysis

## Abstract

Chronic obstructive pulmonary disease (COPD) is one of the leading causes of death worldwide. Current COPD diagnosis (i.e., spirometry) could be unreliable because the test depends on an adequate effort from the tester and testee. Moreover, the early diagnosis of COPD is challenging. The authors address COPD detection by constructing two novel physiological signals datasets (4432 records from 54 patients in the WestRo COPD dataset and 13824 medical records from 534 patients in the WestRo Porti COPD dataset). The authors demonstrate their complex coupled fractal dynamical characteristics and perform a fractional‐order dynamics deep learning analysis to diagnose COPD. The authors found that the fractional‐order dynamical modeling can extract distinguishing signatures from the physiological signals across patients with all COPD stages—from stage 0 (healthy) to stage 4 (very severe). They use the fractional signatures to develop and train a deep neural network that predicts COPD stages based on the input features (such as thorax breathing effort, respiratory rate, or oxygen saturation). The authors show that the fractional dynamic deep learning model (FDDLM) achieves a COPD prediction accuracy of 98.66% and can serve as a robust alternative to spirometry. The FDDLM also has high accuracy when validated on a dataset with different physiological signals.

## Graphical abstract

Chronic obstructive pulmonary disease (COPD) is one of the leading causes of death worldwide, usually associated with smoking and environmental occupational exposures. The COPD detection problem is addressed by constructing two novel COPD physiological signals datasets, demonstrating their complex coupled fractal dynamical characteristics, and performing a rigorous fractional‐order dynamics deep learning analysis to diagnose COPD with high accuracy.

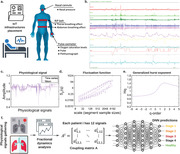

## 1 Introduction

COPD is an increasingly prevalent respiratory disorder, which represents a severe impediment for the quality of life;^[^ ^1^, ^2^ ^]^ it is the third or fourth major cause of death worldwide.^[^ ^3^ ^]^ Medical practice presents COPD as an inflammatory lung condition consisting of a slow, progressive obstruction of airways that reduces pulmonary capacity.^[^ ^4^ ^]^ Medical science has not entirely clarified what triggers COPD; nonetheless, scientists indicate the complex interactions between the environmental factors—such as pollution exposure or smoking—and the genetics^[^ ^5^ ^]^ as likely causes. COPD is not reversible, but early diagnosis creates incentives for achieving a better disease evolution, and an improved patient condition through personalized treatments.^[^ ^6^ ^]^

The Global Initiative for Obstructive Pulmonary Disease (GOLD) defines COPD—based on pulmonary function testing or spirometry—as the ratio between the forced expiratory volume in one second and the forced vital capacity (FEV1/FVC) of <0.7 in a patient with symptoms of dyspnea, chronic cough, and sputum production, with an exposure history to cigarette smoke or biofuels, or occupational particulate matter. The spirometer is a device that measures the lung's volume and air debits, rendered as forced expiratory volume in one second (FEV1), forced vital capacity (FVC), and the ratio between FEV1 and FVC; physicians use these parameters to classify patients in one of the following COPD stages: 1–Mild, 2–Moderate, 3–Severe, and 4–Very Severe. The almost unanimously accepted classification methodology is the COPD Gold Standard,^[^ ^7^, ^8^ ^]^ although there are some differences in applying it.^[^ ^9^ ^]^ Unfortunately, early COPD detection and diagnosis are challenging at the population level because relevant clinical signs are hard to detect in the early phases. When suspected, patients are ordinarily subjected to pulmonary function tests (i.e., spirometry) and mostly diagnosed when they are already in stages 2–4; thus, designing therapies to improve the disease trajectory becomes difficult.^[^ ^10^ ^]^ Another problem with spirometry is that it does not always render reliable results, mainly when not performed in a specialized pulmonary center.^[^ ^11^ ^]^ Nonetheless, the fact that COPD has become a global threat^[^ ^1^, ^2^ ^]^ further emphasizes the importance of decentralizing diagnosis, meaning that finding innovative methods to diagnose COPD outside respiratory medicine centers becomes paramount. Recent medical research suggests that personalized medicine could improve COPD diagnosis.^[^ ^6^ ^]^ One approach to COPD personalized care is identifying patient phenotypes based on comorbidities, simple clinical, and anthropometric data (e.g., age, body‐mass index, smoker status). To this end, the medical practice uses two questionnaires to evaluate symptoms and evaluate the severity of the disease, namely COPD Assessment Test (CAT) and Medical Research Council Breathlessness Scale (MRC).^[^ ^12^, ^13^ ^]^ Also, there are algorithmic methods for clustering COPD patients based on big data, complex network analysis, and deep learning.^[^ ^14^, ^15^, ^16^, ^17^, ^18^ ^]^ However, these techniques have not resulted in high prediction accuracy; the reason is that they only focus on investigating novel machine learning models rather than analyzing the geometric characteristics of the data. Furthermore, big data and Internet‐of‐Things (IoT) solutions were proven to be effective in COPD management, but such existent engineering systems are merely monitoring physiological signals to provide therapeutic feedback to physicians.^[^ ^19^, ^20^ ^]^ Instead, in this paper, in the distribution of moment‐wise estimates of the Hurst exponents in the healthy/COPD groups and offer a rigorous alternative to the conventional (spirometry‐based) methodology for COPD diagnostics. Two hypotheses underpin the solution we introduced:

1. The physiological signals relevant to COPD (e.g., respiratory rate, oxygen saturation, abdomen breathing effort, etc.) have a multi‐fractal nature, and their fractional‐order dynamics specifically characterize the COPD pathogenic mechanisms.
2. We can capture the fingerprints of the COPD‐related physiological processes with the coupling matrix in our mathematical modeling of the physiological dynamics. (In other words, the coupling matrix *A* deciphers the interdependencies and correlations between the recorded signals.)

**Figure 1:**
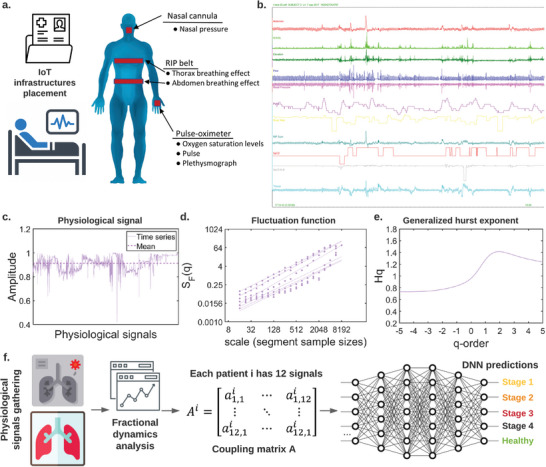
Overview of the proposed method for COPD stage prediction: a) based on the medical observations from the latest research in the field, we identify the physiological signals with relevance in COPD and measure them; b) we record such physiological signals with a medical sensor network—NOX T3™ portable sleep monitor.^[^ ^21^ ^]^ An example of a physiological signal (Abdomen) recorded from a stage 4 COPD patient is shown in (c). d) and e) summarize the multifractal analysis in terms of scaling function and generalized Hurst exponent. [In c–e), we intend to present the case] f) We employ the analysis of the fractional‐order dynamics to extract the signatures of the signals as coupling matrices and fractional‐order exponents and use these signatures (along with expert diagnosis) to train a deep neural network that can identify COPD stages. Reproduced with permission.^[^ ^21^ ^]^ Copyright 2014, Sleep Breath.

In this work, we generate two novel COPD physiological signals datasets (WestRo COPD dataset and WestRo Porti COPD dataset) and implement our method by analyzing the relevant physiological signals recorded with an IoMT (Internet of Medical Things) infrastructure. We extract the fractional dynamics signatures specific to the COPD medical records and train a deep neural network to diagnose COPD stages using both fractal dynamic network signatures and expert analysis (see **Figure** **1**).

## 2 Results

### 2.1 Physiological Signals

#### 2.1.1 Recorded Signals

##### WestRo COPD Dataset

In this dataset, each medical case consists of 12 signal records. First, we recorded seven physiological signals from our patients with the Respiratory Inductance Plethysmography–RIP (signals Thorax Breathing Effort and Abdomen Breathing Effort), the wireless pulse‐oximeter (signals Oxygen Saturation Levels, SpO2 beat‐to‐beat mode, Pulse, and Plethysmograph), and the nasal cannula (signal Nasal Pressure). The NOX T3™ portable sleep monitor integrates and synchronizes the RIP, the wireless pulse‐oximeter, and the nasal cannula. (“Data Collection” in Section 4 provides detailed information). Moreover, the Noxturnal™ software application, which accompanies the NOX T3™ derived five additional signals: RIP Sum (the sum of the abdomen and thorax breathing effort signals), Activity (derived from the X, Y, and Z gravity axes), Position (in degrees, derived from the X, Y, and Z gravity axes, where the supine position is 0 degrees), Flow (derived from the nasal pressure signal), Resp Rate (respirations per minute derived from the RIP Sum signal). All the medical records in this dataset are gathered from four Pulmonology Clinics in Western Romania (Victor Babeş Hospital – VB, Medicover 1 – MD1, Medicover 2 – MD2, and Cardio Prevent – CP clinics).

##### WestRo Porti COPD Dataset

The dataset consists of 6 physiological signals recorded in 13 824 medical cases from 534 individuals during 2013–2020. The patients in the WestRo Porti are screened with the Porti SleepDoc 7 potable PSG device by recording 6 physiological signals (Flow, SpO2, Pulse, Pulsewave, Thorax, Abdomen) overnight. The 6 Porti SleepDoc 7 signals correspond, respectively, to the following NOX T3 signals: Flow, Oxygen Saturation Levels, Pulse, Plethysmograph, Thorax Breathing Effort, and Abdomen Breathing Effort. The reason for involving this dataset in this study is that: 1) we want to involve it as an external dataset to validate our model; 2) we want to test the robustness of our prediction and diagnosis approach where the medical signals records in this dataset interfere with another disease (sleep apnea).

#### 2.1.2 Fractal Properties of Physiological Signals

To verify our first hypothesis, in this section, we show the fractal features of raw signals (Thorax, Oxygen Saturation, Pulse, Plethysmograph, Nasal Pressure, and Abdomen) in healthy persons (stage 0) and critical COPD patients (stage 4) in our WestRo COPD dataset.

As shown in refs. [22, 23, 24, 25], Detrended Fluctuation Analysis (DFA) is an effective method to investigate the statistical scaling and monofractal properties of non‐stationary time series. For instance, the dichotomous models of fractional Gaussian noise (fGn) and non‐stationary fractional Brownian motion (fBm)—initially described by Mandelbrot and van Ness^[^ ^22^ ^]^—have been shown as a proper mono‐fractal modeling framework for physiological signals.^[^ ^23^ ^]^ In addition, DFA is also widely used to investigate the time‐series data in human respiration and heart rate. For instance, Peng et al. applied the DFA technique to quantify the scaling behavior of nonstationary respiratory time series and analyze the presence of long‐range correlations of breathing dynamics in healthy adults.^[^ ^26^ ^]^ Furthermore, Schumann et al. used DFA to measure the autocorrelations in heartbeat intervals and respiration on longer time scales.^[^ ^27^ ^]^ To overcome the challenges of the 'inversed' singularity spectrum in standard multifractal analysis, Mukli, Nagy, and Eke^[^ ^28^ ^]^ proposed the focus‐based multifractal formulas, which compute a moment‐wise global‐error parameter capturing the finite size effects and the signals' degree of multifractality. In order to mine the physiological complexity and account for its nonstationarity, we perform a comprehensive multifractal detrended fluctuation analysis (MF‐DFA) of the collected data.

**Figure 2:**
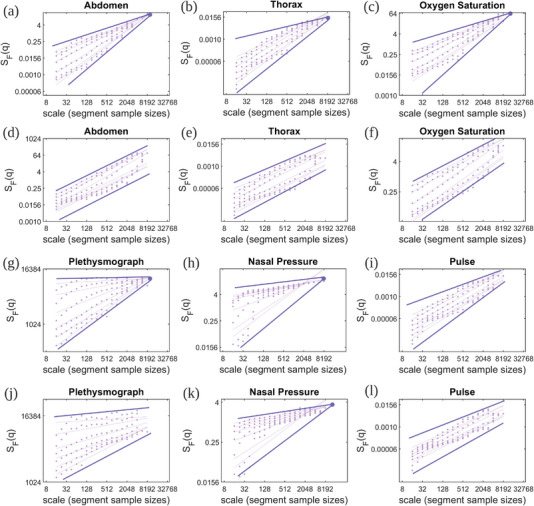
The geometry of fluctuation profiles for the COPD‐relevant physiological signals recorded from a normal abdomen and a stage 4 COPD abdomen. We calculate the scaling functions from 6 raw physiological signals: abdomen, thorax, oxygen saturation, plethysmograph, nasal pressure, and pulse, where the exponents are *q* ∈ [−5, 5]. Panels a–c) and g–i) with signals recorded from a healthy person, d–f) and j–l) with signals recorded from a representative stage 4 COPD patient. The resulting points (pink nodes) of the multifractal scaling function with power‐law scaling will converge to a focus point (dark purple nodes) at the largest scale (*L*) if the tested signal has multifractal features.

To analyze the fractional dynamic characteristics of the COPD physiological processes, we calculate the scaling (fluctuation) functions of the raw signals (Thorax, Oxygen Saturation, Pulse, Plethysmograph, Nasal Pressure, and Abdomen) in healthy people (stage 0) and very severe COPD patients (stage 4) (for detailed information about *MF‐DFA* and the scaling function, see “Multifractal Detrended Fluctuation Analysis” in Section 4). **Figure** **2**a–c,g–i, respectively, show the scaling functions calculated from abdomen, pulse, plethysmograph, thorax, nasal pressure, and oxygen saturation signals generated for a healthy person and Figure 2d–f,j–l illustrate the scaling functions for the same signals from a very severe COPD patient (stage 4). We set the *q* values as *q* ∈ { − 5, −3, −1, 1, 3, 5}. In Figure 2a–c,g–i, we find that the scaling functions under different *q* values will converge to a focus point (the dark purple nodes in each panel), except the Pulse signal. The focus point *S*(*v*, *L*) can be measured as the scaling function's fundamental property (for details on focus point, see “Multifractal Detrended Fluctuation Analysis” in Section 4). Accordingly, if a signal's scaling function has a focus point, it has multifractal features.^[^ ^28^, ^29^ ^]^ The pink lines in Figure 2 are the lines best‐fitted to the scaling function's observed data and the pink dots represent different segmented sample sizes. To be more specific, we show the scaling functions of physiological signals extracted from all stage 4 patients and the healthy people (stage 0) in our dataset (where *q* ∈ [ − 5, 5]) in Supplementary material (for detailed information, see “Hurst Exponents of Physiological Signals” in Supporting Information). Hence, from Figure 2a–c,g–i, the raw signals—except the Pulse signal—in healthy persons have multifractal features. Conversely, in Figure 2d–f,j–l, these scaling functions do not have focus points within the scale (except the Nasal pressure signal), which may suggest that such signals—recorded from severe COPD patients—do not have multifractal features.

**Figure 3:**
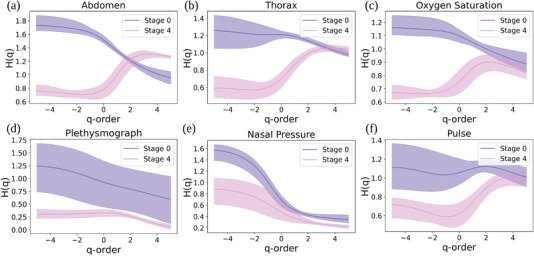
Multifractal analysis of 6 physiological signals from healthy people (stage 0) and stage 4 COPD patients with 95% confidence interval: Generalized Hurst exponent *H*(*q*) as a function of *q*‐th order moments (where *q* values are discretely extracted from −5 to 5) for physiological signals (abdomen (a), thorax (b), oxygen saturation (c), plethysmograph (d), nasal pressure (e), and thorax (f)) extracted from healthy people (stage 0) and severe COPD patients (stage 4).

**Figure** **3** presents the *H*(*q*) comparisons between physiological signals (abdomen (a), thorax (b), oxygen saturation (c), plethysmograph (d), nasal pressure (e), and thorax (f)) extracted from healthy people and patients with 95% confidence interval, where *H*(*q*) is the generalized Hurst exponent and represents the set of associated slopes of the pink lines in Figure 2. As discussed in Bashan et al., the Hurst exponent measures the auto‐correlations of the time‐series data, where the autocorrelations are linked with both short‐term and long‐term memory,^[^ ^30^ ^]^ and larger Hurst exponent values (i.e., *H* > 0.5) represent persistent behavior (i.e., a more correlated structure) in monofractal fractional Gaussian noise type signals.^[^ ^23^, ^31^ ^]^ In contract, the generalized Hurst exponent (*H*(*q*)) can capture the scaling properties of the time series data and reflects the heterogeneous scaling of the *q*th order moments of the increments of the temporal process.^[^ ^28^ ^]^ *H*(*q*) confidence interval curves among healthy people (stage 0) and COPD patients (stage 4) in Figure 3 are not fully overlapped, which shows that the physiological signals extracted from healthy people and severe COPD patients possess different fractional properties (The non‐linear decrease distribution of the *H*(*q*) function illustrates that the fitted lines of scaling functions under different *q* values will converge to a focus point). In Figure 3, all signals recorded from stage 4 COPD patients have different *H*(*q*) confidence intervals than the signals recorded from stage 0 participants, showing that signals collected from patients with different COPD stages have different fractional features. Consequently, the overarching conclusion of Figures 2 and 3 is that the physiological signals with relevance for COPD have different fractional dynamics characteristics in healthy people—on the one hand—and severe COPD patients—on the other hand.

**Figure 4:**
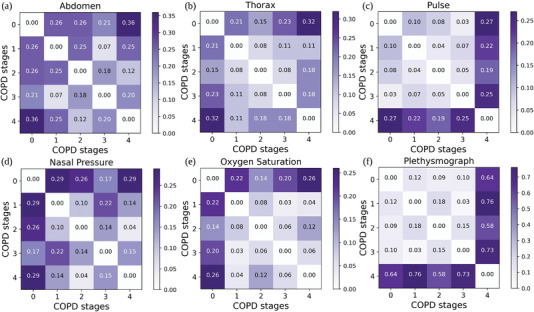
Comparison of Wasserstein distance between the distributions of mean *H*(*q*) curves across different COPD stages (i.e., *H*(*q*) curves in Figure 3, Figure S2, Supporting Information and Figure S3, Supporting Information). Comparison in terms of Wasserstein distance between the *H*(*q*) distribution function for Abdomen (a), Thorax (b), Pulse (c), Nasal Pressure (d), Oxygen Saturation (e), and Plethysmograph (f) signals recorded from patients across all the COPD stages.

To analyze the *H*(*q*) functions across different COPD stages, we calculate the Wasserstein distance between each *H*(*q*) mean value curve in every physiological signal extracted from patients with different stages (we calculate the Wasserstein distances with the wasserstein_distance function in Python's *scipy package* ^[^ ^32^ ^]^). For detailed results about *H*(*q*) curves for all COPD stages, see “Hurst Exponents of Physiological Signals” in Supporting Information. The Wasserstein distance is a metric that measures differences between distributions.^[^ ^33^ ^]^ In **Figure** **4**, we show that, for each physiological signal, the signals' *H*(*q*) function extracted from stage 0 patients have the largest Wasserstein distance from stage 4 patients (except the Plethysmograph signal). In contrast, the *H*(*q*) functions of signals extracted from patients in moderate to severe COPD stages (i.e., 1, 2, and 3) have much smaller Wasserstein distances between each other (except the Abdomen signal). The results presented in Figure 4 represent evidence that reinforces the medical observation stating that it is hard to distinguish early COPD stages. As such, it makes sense to analyze the spatial coupling between these physiological processes (signals) across time. These spatial coupling matrices *A* contain the different fractional features across different signal samples and can help us classify the signals recorded from suspected patients into different COPD stages.

The general conclusion in Figures 2, 3, and 4 is that the physiological signals recorded gathered from healthy individuals and severe COPD patients have distinct fractal properties. (This dichotomy is less evident for the Pulse signal, even if the tendency toward multifractality in healthy individuals is still present.) The notable exception is the Nasal Pressure signal, which has multifractality in both healthy and COPD individuals. This observation suggests that COPD mostly affects the physiology of muscles involved in or supporting breathing (Thorax Breathing Effort and Abdomen Breathing Effort)^[^ ^34^, ^35^ ^]^ and the circulatory system's physiology (reflected in Oxygen Saturation Levels and Plethysmograph).^[^ ^36^ ^]^ Conversely, the upper respiratory tract's physiological dynamics do not appear to be affected even by severe and very severe COPD stages.

### 2.2 Fractional Dynamics Modeling of COPD Relevant Physiological Signals Subject to Unknown Perturbations

The dynamics of complex biological systems possess long‐range memory (LRM) and fractal characteristics. For instance, several recent studies have demonstrated that stem cell division times,^[^ ^37^ ^]^ blood glucose dynamics,^[^ ^38^, ^39^ ^]^ heart rate variability^[^ ^40^, ^41^ ^]^ and brain‐muscle interdependence activity^[^ ^42^ ^]^ are fitted by power‐law distributions.^[^ ^43^, ^44^, ^45^, ^46^ ^]^ The long short‐term memory (LSTM) architecture is one of the most widely used deep learning approaches to analyze biological signals and perform prediction or classification. However, LSTM cannot fully represent the long memory effect in the input, nor can it generate long memory sequences from unknown noise inputs.^[^ ^47^, ^48^ ^]^ Thus, when considering the very long‐time series with long‐range memory, LSTM cannot predict nor classify them with high accuracy. Indeed, in our study, the length of each physiological signal has more than 72 000 data points. We aim to capture both short‐range and long‐range memory characteristics of various physiological processes and—at the same time—investigate the very long COPD signals with high accuracy; therefore, we adopt the generalized mathematical modeling of the physiological dynamics,

(1) $$\begin{array}{ccc} \Delta^{\alpha }x\left[k+1\right] & = & Ax[k]+Bu[k]\\ y[k] & = & Cx[k] \end{array}$$

where $x\in R^{n}$ is the state of the biological system, $u\in R^{p}$ is the unknown input and $y\in R^{n}$ is the output vector.^[^ ^39^ ^]^ The main benefits of this generalized mathematical representation are threefold:

1. The model allows for capturing the intrinsic short‐range memory and long‐range memory of each physiological signal through either an integer or fractional order derivative. To connect the mathematical description with the discrete nature of measurements, the differential operator Δ is used as the discrete version of the derivative; for example, Δ^1^ *x*[*k*] = *x*[*k*] − *x*[*k* − 1]. A differential order of 1 has only one‐step memory, and hence the classic linear‐time invariant models are retrieved as particular cases of the adopted mathematical model. However, when the differential order is 1, the model cannot capture the long‐range memory property of several physiological signals. Furthermore, we write the expansion of the fractional derivative and discretization^[^ ^49^ ^]^ for any *i* ^th^ state (1 ⩽ *i* ⩽ *n*) as
(2) $$\Delta^{\alpha_{i}}x_{i}\left[k\right]=\sum_{j=0}^{k}\psi \left(\alpha_{i},j\right)x_{i}\left[k-j\right]$$
  where α_*i*_ is the fractional order corresponding to the *i* ^th^ state and $\psi \left(\alpha_{i},j\right)=\frac{\Gamma (j-\alpha_{i})}{\Gamma \left(-\alpha_{i}\right)\Gamma \left(j+1\right)}$ with Γ(.) denoting the gamma function. Equation (2) shows that the fractional‐order derivative framework provides a mathematical approach to capture the long‐range memory by including all *x*_*i*_[*k* − *j*] terms.
2. Our modeling approach describes the system dynamics through a matrix tuple (α, *A*, *B*, *C*) of appropriate dimensions. The coupling matrix *A* represents the spatial coupling between the physiological processes across time, while the input coupling matrix *B* determines how the inputs affect these processes. We assume that the input size is always strictly smaller than the state vector's size, that is, *p* < *n*. The coupling matrix *A* plays an essential role in deciphering the correlations between the recorded physiological signals. These correlations (entries of *A*) can indicate different physical conditions. For instance, when probing the brain electrical activity (through electroencephalogram (EEG) signals), the correlations can help at differentiating among various imaginary motor tasks.^[^ ^50^ ^]^ Moreover, as described in this work, we can exploit these correlations to differentiate among pathophysiological states—such as degrees of disease progression—using physiological signals analysis. A key challenge is the estimation accuracy of these correlations (*A* matrix), notably for partially observed data. We have taken care of such limitations by using the concept of unknown unknowns introduced in reference.^[^ ^39^ ^]^
3. Since we may have only partial observability of the complex biological systems, we take care of the unknown stimuli (excitations that may occur from other unobserved processes but cannot be probed); as such, we include in the model the vector variable *u* and study its impact on the recorded dynamics. In essence, we refer to this mathematical model as a multi‐dimensional fractional‐order linear dynamical model with unknown stimuli. The model parameters are estimated using an Expectation‐Maximization (EM) based algorithm described in reference,^[^ ^39^ ^]^ to overcome the lack of perfect observability and deal with possibly small and corrupted measurements. Reference [38] proves that the algorithm is convergent and shows that it reduces modeling errors.

### 2.3 Fractional Dynamics Deep Learning Prediction of COPD stages

After extracting the signals' features (short‐range and long‐range memory) with the fractional dynamic mathematical model, we utilize these features (i.e., coupling matrices *A*) to train a deep neural network to predict patients' COPD stage. Deep learning is a machine learning approach that efficiently combines feature extraction and classification and it is a valuable tool for medical diagnosis (i.e., it can logically explain a patient's symptoms^[^ ^51^ ^]^). We develop the fractional dynamics deep learning model (FDDLM) presented in this section to predict the COPD stages for our WestRo COPD dataset consisting of 4432 medical cases from patients in Pulmonology Clinics from Western Romania. We evaluate these cases and FDDLM by *k*‐fold cross‐validation and hold‐out validation. *K*‐fold cross‐validation is a resampling procedure used to estimate how accurately a machine learning model will perform in practice. In *k*‐fold cross‐validation (*k* = 5), we randomly shuffle the input dataset and split it into 5 disjoint subsets. We select each subset as the test set (20%) and combine the remaining subsets as the training set (80%). In hold‐out validation, we hold one institution out at a time. That is, we hold out data from one institution as a test set, and the remaining data from the other three institutions are used to train the models. The main steps of our approach are:

1. We construct a COPD stage‐predicting FDDLM, and calculate coupling matrix signatures (*A*) of relevant physiological signals (such as Thorax Breathing Effort or Abdomen Breathing Effort, etc.) to be used as the training data.
2. We train FDDLM with our training set to recognize the COPD level based on signal signatures.
3. We test FDDLM with our test set and predict patients' COPD stage.

**Figure 5:**
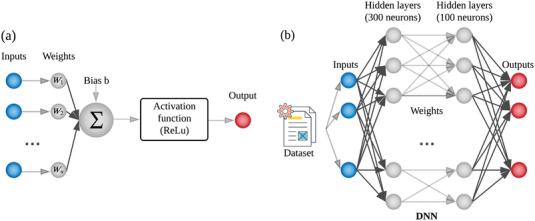
Network formulation for FDDLM: a) Basic structure of an artificial neuron model; b) Overview of the neural network model we trained in FDDLM to identify COPD stages.

Our FDDLM uses a feedforward deep neural network architecture^[^ ^52^ ^]^ with four layers: one input layer, two hidden layers, and one output layer. We present our model's structure in **Figure** **5**. The input layer takes the input (i.e., signal signatures in the coupling matrix *A*) and pass it to hidden layers. From layer to layer, neurons compute the sum of the weighted inputs from the previous layer and settle the results through a nonlinear function (activation function) (see Figure 5a). In our FDDLM, hidden layers' activation functions are rectified linear unit (*ReLU*).^[^ ^53^ ^]^ The *ReLU* activation function returns the input value when the input is ⩽0 (otherwise, it returns 0), that is, *g*(*z*) = *max*(0, *z*) for input value *z*. The output layer's activation function is *softmax* (*S*), which normalizes the input values into a probability distribution. We utilized the *rmsprop* optimizer (with the default learning rate of 0.001) and the categorical cross entropy loss function. To avoid potential overfitting, we insert dropout function (with 0.8 keep rate) after each hidden layer to randomly select and ignore 20% of neurons.

#### 2.3.1 *K*‐Fold Cross‐Validation Results

We process all physiological signals with the fractional dynamical model;^[^ ^39^ ^]^ then, we feed the signal signatures from the coupling matrix *A* to FDDLM. We implement the neural network model in Python with Keras package and executed it on a computer with the Intel Core i7 2.2GHz processor and 16GB RAM.

**Figure 6:**
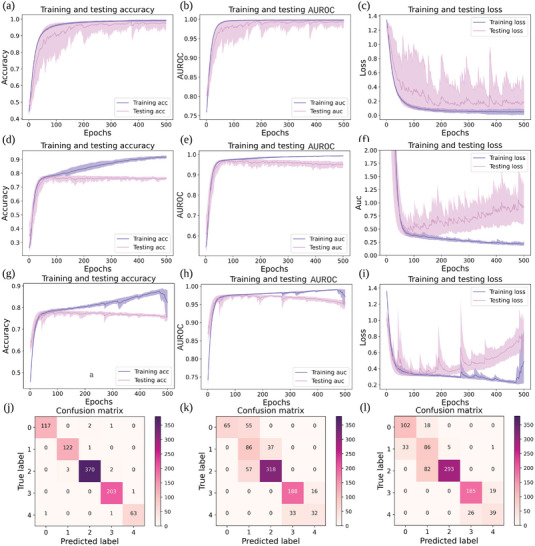
Training and testing result comparisons [accuracy (a,d,g), AUROC (b,e,h), and loss (c,f,i)] of different deep learning models for the *k*‐fold cross‐validation. Training/testing accuracy a), AUROC b), and loss c) for our FDDLM, where the training processes use signal signatures extracted with the fractional dynamic mathematical model. Training/testing accuracy, AUROC, and loss for the Vanilla DNN model d–f) and the LSTM model g–i), where the training processes use the physiological signals recorded with portable sleep monitors (raw data). Both Vanilla DNN and LSTM models share similar network structures and computation parameters with our FDDLM (Vanilla DNN has the same network structure as our model, except the input size). We obtain these results with the *k*‐fold cross‐validation (*k* = 5). We also show the confusion matrices for test set across different models: FDDLM in panel j), Vanilla DNN in k), and LSTM in l).

We evaluate our results based on accuracy, sensitivity, loss, precision, specificity, and area under the receiver operating characteristic curve (AUROC). Our estimation of all results—generated from different models—uses the *k*‐fold cross‐validation method (with *k* = 5). **Figure** **6** presents our model's accuracy, AUROC, and loss curves on training and test sets. We also evaluate our model by comparing it with the Vanilla DNN and LSTM models trained on physiological signals (i.e., raw data) extracted from sleep monitors for reference. The Vanilla DNN and LSTM models have the same hyper‐parameter setting as ours, including the optimizer, dropout configuration, loss function, activation functions, except the input layer size. We aim to choose the classic deep learning models with similar structures as baselines to investigate whether the fractional features (coupling matrix *A*) are easier for models to classify than the raw data. Figure 6a–c shows the training and test results observed from FDDLM. We observe that training and test accuracies and AUROC increase, while loss decreases during training.

Figures 6d–f and 6g–i present the training and test accuracy, AUROC, and loss results of Vanilla DNN model and LSTM model trained with physiological signals (raw data). The training and test results obtained from both Vanilla DNN model and LSTM model display overfitting, as test accuracies decrease (test loss increase) while training accuracies increase (training loss decrease). Thus, to deal with overfitting, we involve the early‐stopping technique^[^ ^54^ ^]^ in choosing the best‐performing Vanilla DNN and LSTM. Compared to our FDDLM, the best‐performing Vanilla DNN and LSTM result in much lower accuracies: 77.72%±0.688% and 78.54%±1.200%, respectively, while our FDDLM achieves 98.66%±0.447%.

Figure 6j–l shows the confusion matrix examples of FDDLM, Vanilla DNN, and LSTM, respectively. The confusion matrices present the prediction results of the test set. (We construct the test set using the last 20% of data in the WestRo COPD dataset and train models with the first 80% of data.)

The results point out that the FDDLM only misclassified 1.35% of the test sets in terms of individual COPD stages. Instead, Vanilla DNN and LSTM models misclassified 24.21% and 21.41% of the test sets, respectively. (We also investigated the possibility of using the convolutional neural network (CNN) model to characterize the physiological signal dynamics obtained from sleep monitors (raw data) and compare it with our FDDLM. The CNN model misclassified 63.87% of the test sets with *k*‐fold cross‐validation. For detailed information about CNN model and results, see “Neural Network Architecture for the WestRo COPD Dataset” in Section 4 and “Training and Testing Results for CNN” in Supporting Information.) **Table** **1** presents the precision, sensitivity, and specificity of our model's predicting results; we find that all these results exhibit a substantial accuracy except the sensitivity of stage 4, which is 96.92%. In conclusion, our FDDLM predicts patients' COPD stages with a much higher accuracy than Vanilla DNN and LSTM models trained with physiological signals (raw data)—without overfitting—and represents an effective alternative to the spirometry‐based diagnostic. (We performed the *K*‐fold analysis of our model's accuracy both on a per‐recording and a per‐patient basis and obtained very similar results; see “Per‐Patient Based *K*‐Fold Analysis” in Supporting Information.)

**Table 1:** The COPD stage predicting results for test set with our Fractional Dynamics Deep Learning Model (FDDLM)

COPD	Stage 0	Stage 1	Stage 2	Stage 3	Stage 4
Sensitivity	97.50%	99.19%	98.66%	99.50%	96.92%
Specificity	99.87%	99.61%	99.41%	99.31%	99.88%
Precision	99.15%	97.60%	99.20%	98.06%	98.44%

#### 2.3.2 Hold‐Out Validation

**Figure 7:**
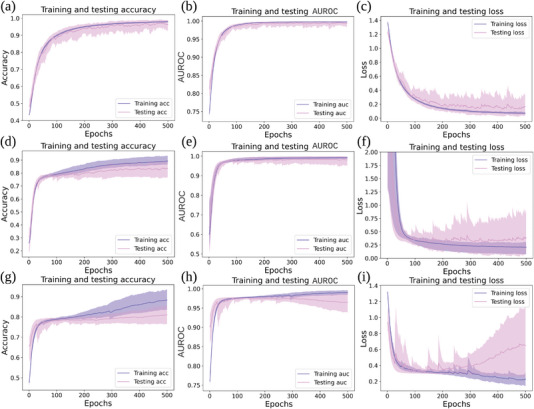
Training and testing result comparisons of different deep learning models for the hold‐out validation. The training/testing accuracy a), AUROC b), and loss c) for our FDDLM, where the training processes use signal signatures extracted with the fractional dynamic mathematical model. The training/test accuracy (d) and (g), AUROC (e) and (h), and loss (f) and (i), for Vanilla DNN model and LSTM model, where the training processes use the physiological signals recorded with portable sleep monitors. Both Vanilla DNN and LSTM models share similar network structures with FDDLM (i.e., same neural network structure but different the input size). We obtain these results by holding out data from every single institution as the test set.

The COPD dataset consists of physiological signals recorded from consecutive patients from four Pulmonology Clinics in Western Romania (Victor Babeş Hospital – VB, Medicover 1 – MD1, Medicover 2 – MD2, and Cardio Prevent – CP clinics). To validate our FDDLM, we hold out all data extracted from a single institution as test set and train models on data recorded from the other three institutions. Following experimental setup from the previous section, we use Vanilla DNN and LSTM models as baselines with hyper‐parameters similar to our FDDLM. **Figure** **7** shows the results of FDDLM, Vanilla DNN, and LSTM, in terms of accuracy, AUROC, and loss curves of training and test sets. We train Vanilla DNN and LSTM models on physiological signals (i.e., raw data). Conversely, we train our FDDLM on the fractional signatures extracted from the raw data.

Figures 7 presents the training and test results (accuracy, AUROC, and loss) generated by FDDLM a–c), Vanilla DNN model d–f), and LSTM g–i) model, respectively. Figure 7a shows that the accuracy of FDDLM increases in training without overfitting. Conversely, the training and testing of Vanilla DNN and LSTM models clearly indicate lower values of the accuracy (80.73%±3.46% and 80.83%±3.67%, respectively) in Figures 7d and 7g, while FDDLM achieves 95.88%±1.76%.

Of note, we observe that the test accuracy under hold‐out validation (95.88%) is lower than the accuracy obtained under *k*‐fold cross‐validation (for more detailed error analysis, we present the visualization of extracted features (embeddings) in the last hidden layer of FDDLM across *k*‐fold and hold‐out validation in the Supporting Information.) The reason for performance degradation in hold‐out is that the data recorded from each medical institution are imbalanced. The Victor Babes (VB) and Cardio Prevent (CP) are two large clinics, and COPD patients are more willing to get diagnosis or medical treatment in large units or hospitals rather than small clinics, especially for severe and very severe COPD patients. Thus, the signals gathered from VB and CP are more comprehensive than the Medicover 1's (MD1) and Medicover 2's (MD2). In hold‐out validation, although we balance the data across different institutions using over‐sampling and under‐sampling approaches, the remaining imbalance in data collection is still the leading cause of the prediction accuracy drop in the hold‐out section.

**Figure 8:**
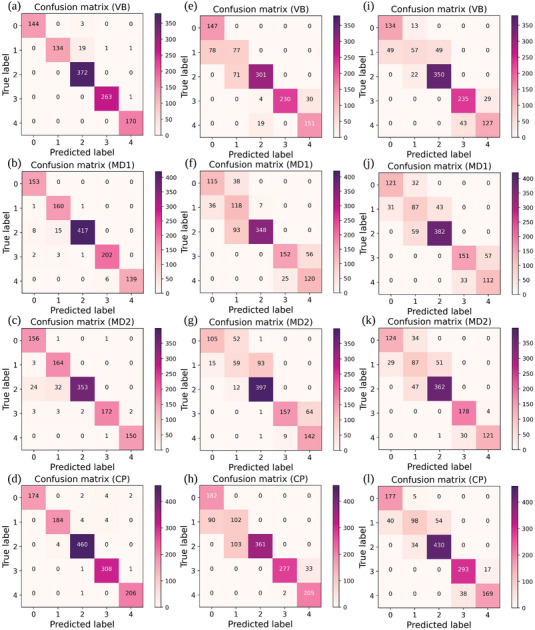
The comparison of confusion matrices resulted from different deep learning models: fractional dynamics a–d), Vanilla DNN e–h), and LSTM i–l). We built the test sets by holding out data gathered from one institution (i.e., VB, MD1, MD2, and CP) at a time. The matrix representations clearly show that our model outperforms both Vanilla DNN and LSTM—in all experiments and for all labels representing COPD stages—in terms of prediction errors.

**Figures** **8**a,d,g,j, 8b,e,h,k and 8c,f,i,l show the confusion matrices (we only present the test set prediction results) for FDDLM, Vanilla DNN, and LSTM models by holding out each institution as the test set, respectively. Results shown in Figure 8 prove that our model outperforms the baselines in prediction accuracy across the entire hold‐out validation process. Especially for the early‐stage detection (i.e., stages 1 and 2), our model achieves a higher accuracy than baselines (for the sensitivity, specificity, and precision of these confusion matrices, see Tables S1–S3, Supporting Information). The reason is that, as opposed to Vanilla DNN and LSTM models, FDDLM can extract the signal signatures that contain the long‐term memory of the time series. We also use the convolutional neural network (CNN) model to characterize the dynamics of the physiological signals recorded with sleep monitors to compare it with our FDDLM; the CNN model misclassified 64.49% of the test sets under hold‐out validation. For detailed information about the CNN model and testing results, see “Neural Network Architecture for the WestRo COPD Dataset” and “Training and Testing Results for CNN” in Supporting Information).

In summary, our model outperforms all baselines in terms of prediction accuracy under both hold‐out and *k*‐fold cross‐validation. The main conclusion is that FDDLM predicts patients' COPD stages with high accuracy and represents an efficient way to detect early COPD stages in suspected individuals. Indeed, such a low‐invasive and convenient tool can help physicians make precise diagnoses and provide appropriate treatment plans for suspected patients.

#### 2.3.3 Transfer Learning

To evaluate our models' performance, we utilize the transfer learning mechanism to investigate the generalizability of our FDDLM. As such, we introduce the WestRo Porti COPD dataset. Transfer learning is a machine learning method that reuses a model designed for analyzing a dataset on another dataset, thus improving the learner from one domain by transferring information from another related domain.^[^ ^55^ ^]^ The medical subjects in the WestRo Porti are consecutive individuals in the Victor Babes hospital records, screened for sleep apnea with the Porti SleepDoc 7 portable PSG device by recording 6 physiological signals; some individuals are also in various COPD stages. (For detailed information, see “Data Collection” in Section 4). The reasons for applying our COPD FDDLM are: 1) we want to verify that our model is valid on an external dataset; 2) we want to test our model's prediction performance when the medical signal records are interfered with by another disease (i.e., sleep apnea).

We test FDDLM with the WestRo Porti COPD dataset to check the prediction performance. Since the WestRo COPD Porti dataset only have 6 signals (whereas our model uses 12 signals), we reconstructed the input size of the models from 144 × 1 to 36 × 1, retrained a new FDDLM with WestRo and tested it on the WestRo Porti COPD dataset to check the performance. (Note that the WestRo Porti COPD dataset patients are not included in the WestRo COPD dataset). The prediction accuracy of FDDLM is 90.13%± 0.89% with fine‐tuning. The explanation for the accuracy drop is that i) the models are previously designed for analyzing medical records with 12 signals, not 6; ii) the two datasets are recorded by two different portable devices having different frequencies, which influences the convergence of the coupling matrices; iii) the co‐existed sleep apnea in the medical records gathered from the WestRo Porti COPD dataset also influence the prediction performance.

#### 2.3.4 Summary

Nowadays, DNN and LSTM models are the two popular deep learning models in analyzing and classifying time‐series data. However, they do not present high‐performance when the time series are precisely long (the reason is that the current model cannot correctly extract the long‐term memory from very long time series). In this work, we developed a novel fractional dynamics‐based model which can appropriately analyze long‐term memory from COPD physiological signals datasets by extracting fractional features (coupling matrix *A*) from very long time‐series data. The extracted fractional features are more straightforward for deep learning models to classify than the raw signals, and even the linear classifier achieves a good accuracy (for detailed information, see “Linear Classifier of Coupling Matrices” in Supporting Information). Therefore, based on the results shown in *k*‐fold cross‐validation, hold‐out validation, and external validations, we conclude that our FDDLM has enough generalizability to be applied to different kinds of COPD records that contain physiological signals. Indeed, based on the transfer learning results, we argue that our FDDLM is robust enough to predict COPD stages across different datasets with high accuracy.

Besides the high accuracy of our COPD stage prediction method, we made a deeper analysis of the cases where our predictions failed. Overall, we have 19 misclassified cases (out of 534); most of them (i.e., 9) correspond to borderline cases where the spirometry values are exactly (or very close to) threshold values between stages. Some borderline COPD cases also overlap with other respiratory diseases, such as sleep apnea or asthma (3 cases), while one borderline case overlaps with heavy smoking. We also found that 7 misclassified cases overlap with comorbidities, such as severe sleep apnea, asthma, or obesity. For 3 cases, there is no apparent explanation for the misclassification, although one of them is a heavy smoker; in all these 3 cases, the individuals have no COPD but are predicted at stage 2. Another finding is that the noise in physiological signals recordings may rarely cause misclassifications of non‐COPD cases (i.e., stage 0), as we noticed in 3 cases (in our datasets, we have 143 patients with stage 0).

The overarching conclusion is that comorbidities (especially sleep apnea and asthma) can alter the physiological signals to affect the prediction accuracy, mainly when dealing with borderline COPD stages. Future works have to consider the comorbidity cases carefully. Indeed, more people in the aging population suffer from multi‐morbidity, defined as two or more chronic conditions. COPD is common in multi‐morbid patients, and many patients with COPD present concomitant other obstructive diseases, such as obstructive sleep apnea (OSA) and asthma, due to an increased prevalence of obesity, smoking, and allergy in the general population.^[^ ^56^ ^]^ Recent estimation of OSA prevalence shows almost 1 billion people affected, with prevalence exceeding 50% in some countries.^[^ ^57^ ^]^ Moreover, around 300 million people have asthma worldwide, and it is likely that by 2025 a further 100 million may be affected.^[^ ^58^ ^]^

## 3 Discussion

COPD is often a silent and late‐diagnosed disease, affecting over 300 million people worldwide; intrinsically, its early discovery and treatment are crucial for the patient's quality of life and—ultimately—survival.

The inception of a disease entails a preclinical period where it is asymptomatic and—perhaps—reversible; ideally, this period includes very early events that can occur even before birth.^[^ ^59^ ^]^ Early COPD stages do not exhibit evident clinical signs; therefore, conventional spirometry‐based diagnosis becomes improbable. However, the development of biomarkers that include detecting genetic variants for COPD development's susceptibility is a priority. The COPD onset is a phase of early COPD where the disease may express itself with some symptoms, including a minimal airflow limitation.^[^ ^60^ ^]^ In this phase, spirometry is insufficient to attain a reliable diagnosis, which calls for new COPD detection tools.

The current strategy of waiting for surfacing symptoms to signal the disease presence is not efficient if we want to impact COPD's natural course. Targeting early COPD stages in younger individuals could identify those susceptible to rapid disease progression, leading to novel therapies to alter that progression. New validated biomarkers (other than spirometry) of different lung function trajectories will be essential for the design of future COPD prevention and treatment trials.^[^ ^61^ ^]^ Indeed, spirometry with FEV1 may not be the most sensitive test and may have particular limitations in identifying the early COPD stages. Moreover, impulse oscillometry and specific airway conductance were able to identify more subtle changes in lung function than traditional spirometry.^[^ ^62^ ^]^ Impulse oscillometry can identify abnormalities in patients who report COPD symptoms but do not have abnormal spirometry.^[^ ^63^ ^]^ Such complementary diagnostic modalities could potentially aid in the early recognition of COPD, especially those whose symptoms do not match their spirometry results.

The adjustment of current diagnostic approaches and the adoption of alternative modalities may allow for earlier identification of COPD patients. The period of the most rapid decline in lung function occurs early, and during this period, different testing strategies, smoking cessation efforts, and the initiation of treatment may be most beneficial.

This work proposes an alternative, precise diagnostic approach to overcome the conventional (spirometry‐based) method's limitations by using a fractional dynamics deep learning methodology. We involved the fractional‐order dynamics model in extracting the signatures from the physiological signals (recorded by a medical sensor network from suspected patients) and trained a neural network model with these signal signatures to make the COPD stage prediction. From a clinical standpoint, our fluctuation profile analysis for physiological signals with relevance in COPD (see Figure 2) shows that multifractality is the fingerprint of healthiness—in healthy people, physiological signals present both short and long‐range memory. Conversely, monofractals or narrow‐spectrum multifractals indicate a medical condition. We noticed two exceptions to this observation; first, the Pulse signal is narrow‐spectrum multifractal even in non‐COPD (i.e., stage 0) individuals; second, the Nasal Pressure signal is multifractal in both COPD and non‐COPD subjects. The possible explanation for the fact that the Pulse signal is not entirely multifractal in non‐COPD individuals is that—most probably—such subjects may have other medical conditions, such as sleep apnea or cardiovascular problems, which (along with the associated medication) altered the short and long‐range properties of the signal. (Indeed, all patients were referred for polysomnography because of suspicion of respiratory disorders; some turned out to be COPD‐free, yet most of them have other respiratory disorders, such as sleep apnea, as well as cardiovascular conditions.) The Nasal Pressure signal is multifractal in both COPD and non‐COPD subjects because it manifests upper airway dynamics, which may be less affected as COPD is an inflammation (resulting in the narrowing) of the lung airways.

We confirm the results with *k*‐fold cross‐validation and hold‐out validation and show that our approach can predict the patients' COPD stages with high accuracy (98.66%± 0.45%). The accuracy is particularly high for COPD stages 1–3, suggesting that our method is distinctly efficient for detecting early‐stage COPD. Furthermore, based on the transfer learning validation, we prove that our model can also achieve high prediction accuracy when the medical signal records are interfered with by another disease (i.e., sleep apnea). Our work makes two main contributions in medical diagnosis and machine learning fields. First, our fractional dynamics deep learning model makes a precise and robust COPD stage prediction that can work before the disease onset, making it especially relevant for primary care in remote areas or geographical regions missing medical experts, where the vast majority of patients with early and mild COPD are diagnosed and treated (Although the fractional‐order dynamic model performs well in diagnosing COPD, it may not be generalized in investigating other physiological signals. Indeed, not all physiological signals have multifractal features (e.g., Ivanov et al. showed that the human gait interstride interval time series among healthy people do not show multifractality^[^ ^64^ ^]^)). Second, we developed a valid fractional deep learning approach that outperforms the traditional deep learning model (e.g., DNN, LSTM, CNN) of classifying and analyzing very long time‐series raw data. (We provide detailed information to explain why our model can efficiently reduce the learning complexity and achieve a high prediction accuracy in “Mutual Information Analysis” in Section 4).

Nowadays, the conventional spirometry‐based diagnosis is the dominating approach to diagnosing COPD. The problem is that it entails many error‐prone steps/stages involving human intervention such that general practitioners or well‐trained nurses may also misdiagnose suspected patients (of the 4610 subjects, 96.5% had a valid screening spirometry test^[^ ^65^ ^]^). Such a result emphasizes that training and technique reinforcement are paramount, yet many primary care units do not have the resources to perform them. In this paper, our fractal dynamics deep learning method eliminates human intervention (and error) as much as possible; any nurse or MD can place the sensors on the patient's body, turn on the NOX device to record the physiological signals in its local memory. Afterward, we are dealing with a completely automated, computer‐based process. The sufficient signal length required for a correct diagnostic is 10 min (for detailed information, see “Convergence of Coupling Matrix”). Therefore, our method is simple, robust, requires little human intervention, and has a relatively small duration of physiological signal records; this also makes it suitable for addressing critical social aspects of healthcare. First, there is equal opportunity in accessing reliable medical consultation for COPD, especially in areas with a lower socioeconomic status where people do not have the means to travel to a specialized state‐of‐the‐art respiratory clinic.^[^ ^66^ ^]^ With our method, any medical mission in such an area can efficiently record data from many individuals in need and then process it automatically. Second, our method abides by the commandments of universal health care amid the COVID‐19 pandemic, as it filters most of the physical interaction entailed by regular spirometry.^[^ ^16^ ^]^ Although the MF‐DFA methods we use are widely used, they cannot exclude that the focus points are due to bimodality, multifractal noise, or mere monofractality. Hence, in future work, we plan to employ the robust multifractal analysis developed by Mukli et al.^[^ ^28^ ^]^ to analyze the physiological signals and develop a new machine‐learning framework to improve the robustness of predicting COPD stages.

## 4 Experimental section

### Data Collection—WestRo COPD dataset

The study cohort represents consecutive patients from 4 Pulmonology Clinics in Western Romania (i.e., the WestRo cohort, comprising patients from Victor Babeş – VB, Medicover 1 – MD1, Medicover 2 – MD2, and Cardio Prevent – CP clinics). Data consist of physiological signals recorded over long periods (i.e., 6‐24 hours), using a protocol that ensures complete patient privacy. To obtain a reliable medical diagnostic for each patient, the following data records were also collected: age, sex, body mass index (BMI, as a ratio between mass in kilograms and the squared value of height in meters), smoking history (in years since quitting smoking, with value 0 representing current smokers), FVC and FEV1 in liters and percentage (used to render the COPD stage diagnosis according to the ERS/ATS recommendation,^[^ ^67^ ^]^ with stage 0 representing no COPD), COPD assessment test (CAT) and dyspnea severity with modified Medical Research Scale (MRC) questionnaires, exacerbations (number of moderate to severe exacerbation in the last year), COPD onset (number of years since the onset). For detailed information about CAT and MRC, see “Standard Questionnaires, Exacerbation History, and Comorbidities of COPD Patients” in Supporting Information. All data about body mass index (BMI), COPD onset, standard questionnaires (CAT – COPD assessment test, mMRC – modified Medical Research Council dyspnea scale), exacerbation history, and comorbidities (cardiometabolic, cancer, metabolic, psychiatric, renal) for all the patients in our dataset in **Table** **2**.

**Table 2:** Essential information of all the COPD patients in our dataset which include medical center; COPD stage; COPD onset; age; gender; smoking status; body mass index (BMI); standard questionnaires (CAT—COPD assessment test, mMRC—modified Medical Research Council dyspnea scale), exacerbation history, and comorbidities (cardiometabolic (CC), cancer (CA), metabolic (MC), psychiatric (PC), and renal (RD))

Patients ID	Center	COPD stage	COPD onset	Age	Gender	Smoking status	BMI	CAT	MRC	Exacerbation	CC	CA	MC	PC	RD
P1	CP	2	0	66	M	Ex 7	43.27	26	3	1	1	0	1	0	1
P2	CP	2	1	63	F	Smoker	48.49	33	3	1	1	0	1	0	0
P3	CP	2	0	43	M	Smoker	33.58	24	3	0	1	0	0	0	0
P4	CP	2	7	71	M	Ex 11	27.21	22	2	0	1	0	1	0	0
P5	CP	3	0	63	M	Ex 15	47.12	22	2	1	1	0	1	0	0
P6	VB	3	7	70	M	Ex 12	27.31	22	2	1	1	0	0	0	0
P7	MD1	2	4	72	M	Ex 23	68.17	24	3	1	1	0	1	0	0
P8	VB	2	2	88	M	Ex 32	42.97	32	3	1	1	0	1	0	1
P9	CP	2	12	54	M	Smoker	21.91	23	2	2	1	0	0	0	0
P10	MD2	2	0	67	M	Ex 10	31.14	21	3	0	1	0	1	0	0
P11	CP	2	0	66	M	Ex 12	48.45	32	3	1	1	0	1	0	1
P12	VB	3	5	76	M	Ex 8	28.91	34	4	1	1	0	1	0	1
P13	CP	3	7	70	M	Ex 25	36.57	26	3	0	1	0	0	0	0
P14	MD1	3	1	64	M	ex 24	26.5	15	2	0	1	0	0	0	0
P15	VB	2	5	59	M	Smoker	38.1	31	3	1	1	0	1	0	0
P16	VB	1	1	48	F	ex 15	28.4	13	2	1	0	0	1	0	0
P17	CP	1	2	54	M	ex 10	26.3	11	2	0	1	1	0	0	0
P18	VB	1	1	61	M	ex 21	31.5	13	2	1	1	0	1	0	0
P19	CP	2	0	62	M	Smoker	23.59	16	2	0	1	0	0	0	0
P20	VB	2	0	63	M	Smoker	32.81	14	2	1	1	0	0	0	0
P21	VB	4	5	64	M	Smoker	32.87	28	3	2	1	1	0	0	0
P22	CP	2	0	76	M	Smoker	35.43	36	4	1	1	0	0	0	0
P23	VB	1	2	69	M	Ex 22	37.35	28	3	0	1	0	1	0	0
P24	CP	2	1	70	F	no	44.1	25	2	1	1	0	1	0	0
P25	VB	3	0	72	M	Ex 10	39.86	36	4	1	1	0	1	0	1
P26	MD2	3	1	65	M	Ex 1	28.4	24	2	0	1	0	1	0	1
P27	CP	1	3	56	M	ex 5	34.1	19	2	0	0	0	1	0	0
P28	CP	3	0	75	M	Smoker	23.67	32	3	1	1	0	0	0	0
P29	VB	3	3	60	M	Smoker	22.15	21	3	0	0	0	0	1	0
P30	CP	3	2	55	F	Smoker	39.1	27	2	2	1	0	0	0	0
P31	MD2	4	6	67	M	Ex 2	37.18	34	4	1	1	1	1	1	0
P32	CP	4	7	64	M	Ex 4	33.26	28	3	0	1	0	1	0	0
P33	VB	2	3	58	M	Smoker	27.08	26	3	1	1	0	0	1	0
P34	CP	2	3	53	M	Ex 38	29.67	16	2	1	1	0	0	0	0
P35	VB	3	1	76	M	Ex 6	36.39	32	3	1	1	0	0	0	0
P36	CP	4	8	40	M	no	41.5	35	3	2	1	0	0	1	0
P37	CP	2	0	77	M	Ex 23	38.06	26	3	0	1	0	1	0	0
P38	MD1	2	1	49	F	no	40.2	16	2	0	0	0	0	0	0
P39	CP	2	0	67	M	Ex 7	22.89	28	3	1	1	0	1	0	0
P40	CP	3	8	68	F	Ex 7	22.86	28	3	3	1	0	0	0	0
P41	VB	2	3	72	M	Ex 20	31.05	19	2	1	1	0	1	1	0
P42	CP	3	12	67	M	Ex 9	30.42	29	2	1	1	0	0	0	0
P43	CP	3	0	68	M	Smoker	33.95	34	3	1	1	1	1	0	0
P44	VB	4	1	65	F	Smoker	32.46	18	2	0	1	0	0	0	0
P45	VB	2	3	56	M	Smoker	18.62	12	2	2	0	0	0	0	0
P46	VB	2	2	43	M	no	31	17	2	1	0	0	1	0	0
P47	CP	2	1	55	F	ex 10	28.5	20	2	0	1	0	0	0	0
P48	CP	0	0	65	M	no	33	0	0	0	0	0	0	0	0
P49	MD1	0	0	43	M	no	35.9	0	0	0	0	0	0	0	0
P50	MD2	0	0	68	M	ex 24	31.4	0	0	0	0	0	0	0	0
P51	MD1	0	0	46	M	no	31.3	0	0	0	0	0	0	0	0
P52	MD1	0	0	51	M	no	25.4	0	0	0	0	0	0	0	0
P53	VB	0	0	72	F	Smoker	27.4	0	0	0	0	0	0	0	0
P54	MD2	0	0	57	M	ex 15	29.6	0	0	0	0	0	0	0	0

### WestRo Porti COPD dataset

The WestRo Porti cohort consists of polysomnography (PSG) physiological signals recorded in 13824 medical cases from 534 individuals during 2013–2020. The subjects in the WestRo Porti are consecutive individuals in the Victor Babes hospital records, screened for sleep apnea with the Porti SleepDoc 7 portable PSG device by recording 6 physiological signals (Flow, SpO2, Pulse, Pulsewave, Thorax, Abdomen) overnight, during sleep. The 6 Porti SleepDoc 7 signals correspond, respectively, to the following NOX T3 signals: Flow, Oxygen Saturation Levels, Pulse, Plethysmograph, Thorax Breathing Effort, and Abdomen Breathing Effort.

In this work, the same medical doctor gave all diagnoses that led to determining the COPD labels across all institutions. Moreover, the medical doctor used the same devices and diagnosis method (sensors to collect physiological signals from patients and spirometers). In addition, the same medical doctor collected the data in all clinics; spirometry was conducted with the help of trained, experienced technicians, certified in pulmonary function testing, following the ATS/ERS protocol (American Thoracic Society/European Respiratory Society). In all clinics included in our study, there is a quality control program for all procedures.

Spirometry quality assurance includes examining test values and evaluating both the volume‐time and flow‐volume curves for evidence of technical errors. During testing, technicians record a valid test composed of at least 3 acceptable maneuvers with consistent (i.e., repeatable) results for FVC and FEV1. Achieving repeatability during testing means that the differences between the largest and second‐largest values for both FVC and FEV1 are within 150 mL. Additional maneuvers can be attempted—up to a maximum of 8—to meet these criteria for a valid test. The observer bias is reduced by ensuring that observers are well trained (specialized clinics do that regularly with certification diplomas), having clear rules and procedures in place for the experiment (i.e., the ERS/ATS protocol), and ensuring that behaviors are clearly defined. Therefore, since the same medical doctor performed all evaluations with the same equipment and diagnosis approach, the authors are confident that they substantially mitigated the intra‐ and inter‐observer variability.

### Multifractal Detrended Fluctuation Analysis

Multifractal detrended fluctuation analysis (MF‐DFA) is an effective approach to estimate the multifractal properties of biomedical signals.^[^ ^28^ ^]^ The first step of MF‐DFA is to calculate the cumulative profile (*Y*(*t*))

(3) $$Y\left(t\right)=\sum_{i=1}^{t}X\left(i\right)-\left\langle X\left(i\right)\right\rangle$$

where *X* is a bounded time series. Then, divide the cumulated signal equally into *N*_*s*_ non‐overlapping time windows with length *s*, and remove the local line trend (local least‐squares straight‐line fit) *y*_*v*_ from each time window. Therefore, *F*(*v*, *s*) characterizes the root‐mean‐square deviation from the trend (i.e., the fluctuation),

(4) $$F\left(v,s\right)=\sqrt{\frac{1}{s}\sum_{i=1}^{s}{\left\{Y\left\lfloor \left(v-1\right)s+i\right\rfloor -y_{v}\left(i\right)\right\}}^{2}}$$

In ref. [28], the authors defined the scaling function as

(5) $$S\left(q,s\right)={\left\{\frac{1}{N_{s}}\sum_{v=1}^{N_{s}}{\mu (v,s)}^{q}\right\}}^{1/q}$$

where μ is an appropriate measure which depends on the scale of the observation (*s*). Hence, the scaling function is defined by substituting Equation (4) into Equation (5),

(6) $$S_{F}\left(q,s\right)={\left\{\frac{1}{N_{s}}\sum_{v=1}^{N_{s}}{\left\{\frac{1}{s}\sum_{i=1}^{s}{\left\{Y\left\lfloor \left(v-1\right)s+i\right\rfloor -y_{v}\left(i\right)\right\}}^{2}\right\}}^{q/2}\right\}}^{1/q}$$

The moment‐wise scaling functions for a multifractal signal exhibit a convergent structure that yields to a focus point for all *q*‐values. The convergent structure was first introduced in ref. [28], and such focus points, as described in ref. [28], can be deduced from Equation (5), by considering the signal length as a scale parameter,

(7) $$S\left(q,L\right)={\left\{\frac{1}{N_{s}}\sum_{v=1}^{N_{L}}{\mu (v,L)}^{q}\right\}}^{1/q}={\left\{\mu {\left(v,L\right)}^{q}\right\}}^{1/q}=\mu \left(v,L\right)$$

where the value of μ represents the entire signal, namely *N*_*L*_ = 1 (i.e., takes only one time window into consideration). According to Equation (7), the scaling function *S*(*q*, *L*) becomes independent from the exponent *q* and the moment‐wise scaling functions will converge to μ(*v*, *L*) which is the mathematical definition of the focus point.

### Neural Network Architecture for the WestRo COPD Dataset—Fractional dynamics deep learning model (FDDLM)

In our work, FDDLM consists of two parts: 1) fractional signature extraction (for more details, please see “Multifractal Detrended Fluctuation Analysis”) and 2) a deep learning model. Keeping in mind the input size of our training data (i.e., the coupling matrix *A*) and available GPU computational power, a deep neural network (DNN) architecture was constructed to handle the training and prediction progress. The network was built with the TensorFlow Python framework.^[^ ^68^ ^]^ The deep neural network consists of 6 layers: 1 input layer, 2 hidden layers, 2 dropout layers, and 1 output layer. Also, we resampled the input data (matrix *A*) to 144 × 1 voxels and normalized each value within the range [0, 1] (normalization is a technique for training deep neural networks that standardizes the inputs to a layer). We placed the dropout layers after each hidden layer with a 20% drop rate (the first hidden layer has 300 neurons and the second hidden layer has 100 neurons); each fully connected hidden layer utilizes the *ReLU* activation function. The *softmax* is utilized as the activation function in the output layer. The DNN is optimized with the *rmsprop* optimizer with a learning rate of 0.0001 and trained with the *crossentropy* loss function. FDDLM is trained over 500 epochs with a batch size of 64 samples. Overall, the number of trainable parameters of the deep learning model is 74 105.

### Vanilla deep neural network (DNN) model

The Vanilla DNN model shares the same network structure with the deep learning model in our FDDLM, except the input layer. The Vanilla DNN contains 6 layers: 1 input layer (the input data, namely, the physiological signals are reshaped to 72000 × 1 voxels, and each value is normalized within the range [0, 1]), 2 hidden layers (the first hidden layer has 300 neurons and the second hidden layer has 100 neurons), 2 dropout layers, and 1 output layer. The activation function for each fully connected hidden layer is *ReLU*, and the activation function for the output layer is *softmax*. The Vanilla DNN model is optimized with the *rmsprop* optimizer, having a default learning rate of 0.0001, and trained with the *crossentropy* loss function. The model is trained over 500 epochs with a batch size of 64 samples. The total number of trainable parameters of the Vanilla DNN model is 21,630,905.

### Long short‐term memory (LSTM) model

The LSTM model in this work has the following layers: an input layer (the input physiological signals are reshaped to 6000 × 12 voxels, and each value is normalized within the interval [0, 1]), an LSTM layer (with 300 neurons), a dropout layer (with a 0.2 dropout rate), a dense layer (with 100 neurons), a dropout layer (with a 0.2 dropout rate), and an output layer. *ReLU* is the activation function for the LSTM and dense layers. The model is optimized with *rmsprop* having a default learning rate of 0.0001 and trained with the *crossentropy* loss function. The LSTM model is trained over 500 epochs with a batch size of 64 samples. The total number of trainable parameters of the LSTM model is 535 805.

### Convolutional neural network (CNN) model

The CNN model in this paper has the following layers: an input layer (the input physiological signals are reshaped to 72 000× 1 voxels, each value normalized within the range [0, 1]), a convolutional layer (64 neurons), a flatten layer, a dropout layer (with a 0.2 dropout rate), a dense layer (with 32 neurons), a dropout layer (with a 0.2 dropout rate), and an output layer (with 5 neurons). *ReLU* is the activation function for the convolutional and dense layers, while *softmax* is the activation function for the output layer. The CNN model is optimized with *rmsprop* having a default learning rate of 0.0001 and trained with the *crossentropy* loss function. The CNN model is trained over 500 epochs with a batch size of 64 samples; the total number of trainable parameters is 147,456,453.

**Figure 9:**
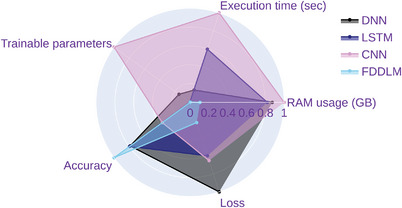
The radar plot for measuring the complexity and prediction performance for the WestRo COPD dataset across different deep learning models under *k*‐fold validation (*k* = 5): fractional‐dynamics deep learning model (FDDLM), Vanilla deep neural network (DNN), long short‐term memory (LSTM), and convolutional neural network (CNN). We normalized all the values represented in this plot.

Resource usage and performance across different models under *k*‐fold cross‐validation are further compared—namely, FDDLM, Vanilla DNN, LSTM, and CNN—by measuring the following metrics: execution time, trainable parameters, RAM usage (in GB) and accuracy. The evaluation results are shown in **Figure** **9** and **Table** **3** (Of note, the values in Table 3 are the mean results under *k*‐fold validation (*k* = 5) and the values in Figure 9 are the normalized results from Table 3). For Figure 9 and Table 3, it is observed that the FDDLM has the highest accuracy (i.e., 96.18%) with the lowest complexity (i.e., memory usage and execution time). These findings indicate that our model's predictions are more accurate while requiring a lower complexity than traditional machine learning models (i.e., Vanilla DNN, CNN, and LSTM).

**Table 3:** Complexity and prediction performance for the WestRo COPD dataset across different deep learning models under *k*‐fold validation (*k* = 5): fractional dynamics deep learning model (FDDLM), Vanilla deep neural network (DNN), long short‐term memory (LSTM), and convolutional neural network (CNN)

	FDDLM	DNN	LSTM	CNN
RAM usage (GB)^b)^	**0.98**	8.17	7.68	9.35
Execution time (sec)^b)^	**1076**	47 590	205 135	345 085
Trainable parameters^b)^	**74 105**	21 630 905	535 805	147 456 453
Test accuracy (%)^a)^	**98.66**	77.72	78.54	36.12
Loss^b)^	**0.2170**	0.9601	0.5728	0.6245

^a)^ indicates higher;

^b)^ lower values are better. All results are evaluated on the same machine for fair comparison.

### Challenges and Limitations of Spirometry in COPD

Spirometry is a physiological test that measures the maximal air volume that an individual can inspire and expire with maximal effort, thus assessing the effect of a disease on lung function. Together with the medical history, symptoms, and other physical findings, it is an essential tool that provides essential information to clinicians in reaching a proper diagnosis.^[^ ^69^ ^]^ Indeed, standard spirometry is a laborious procedure: it needs preparation, a bronchodilation test, performance assurance, and evaluation.^[^ ^70^ ^]^

### Preparation

1) The ambient temperature, barometric pressure, and time of day must be recorded. 2) Spirometers are required to meet International Organization for Standardization (ISO) 26782 standards, with a maximum acceptable accuracy error of ±2.5%. 3) Spirometers need calibration daily, with calibration verification at low, medium, and high flow. 4) The technicians have to make sure that the device produces a hard copy of the expiratory curve plot to detect common technical errors. 5) The pulmonary function technician needs training in the optimal technique, quality performance, and maintenance. 6) There are activities that patients should avoid before testing, such as smoking or physical exercise. 7) Patients should be adequately instructed and then supported to provide a maximal effort in performing the test to avoid underestimating values and ultimately diagnosis errors.

### Bronchodilation

1) The forced expiratory volume in one second (FEV1) should be measured 10–15 min after the inhalation of 400 mcg short‐acting beta2 agonist, or 30–45 min after 160 mcg short‐acting anticholinergic, or the two combined.^[^ ^71^ ^]^ 2) Physicians also developed new withholding times for bronchodilators before bronchodilator responsiveness testing.^[^ ^69^ ^]^

### Performance assurance

1) Spirometry should be performed using standard techniques. 2) The expiratory volume/time traces should be smooth and without irregularities, with *a* less than 1 s pause between inspiration and expiration. 3) The recording should be long enough to reach a volume plateau; it may take more than 15 ss in severe cases.^[^ ^72^ ^]^ 4) Both forced vital capacity (FVC) and FEV1 should represent the biggest value obtained from any of three out of a maximum of eight technically good curves, and the values should vary by no more than 5% or 150 mL—whichever is bigger.^[^ ^73^ ^]^ 5) The FEV1/FVC ratio should be taken as the technically acceptable curve with the largest sum of FVC and FEV1.^[^ ^74^ ^]^

### Evaluation

1) The measurements evaluation compares the results with appropriate reference values—specific to each age, height, sex, and race group. 2) The presence of a post‐bronchodilator FEV1/FVC < 0.70 confirms the presence of airflow limitation.^[^ ^75^ ^]^

It is clear that the diagnosis process—primarily relying on spirometry—is pretty complex and, thus, prone to errors because of human intervention. The large university clinics, such as our Victor Babes clinic in Timisoara (and the other institutions included in our paper's recordings), avoid errors by carefully training their personnel and enforcing strict procedures. Additionally, experienced and well‐trained physicians corroborate the spirometry results with other clinical data, such that diagnostic mistakes are highly improbable. However, a big problem is faced with spirometry in primary care offices, which do not have all resources to consistently abide by the quality assurance steps (preparation, bronchodilation test, performance assurance, and evaluation). Hegewald ML et al. showed that most spirometers tested in primary care offices were not accurate, and the magnitude of the errors resulted in significant changes in the categorization of patients with COPD. Indeed, they obtained acceptable quality tests for only 60% of patients.^[^ ^76^ ^]^ In a similar study, the authors reported a spirometry accuracy varying from 69.1% to 81.4% in the primary care offices.^[^ ^77^ ^]^ These prior experimental studies and findings are significant for the medical community and constitute the motivation for our paper since primary care offices have an essential role in the early detection of COPD cases.

### Definition of COPD Stages

The diagnosis of COPD is based on persistent respiratory symptoms such as cough, sputum production, and dyspnea, together with airflow limitation (caused by significant exposure to smoking, noxious particles, or gases) evaluated with spirometry. The labels or disease stages are defined in the standard guideline of the worldwide medical community.^[^ ^78^ ^]^ Based on the FEV1 (forced expiratory volume in one second) value measured by spirometry, the Global Initiative for Chronic Obstructive Lung Disease (GOLD) guideline system categorizes airflow limitation into stages. In patients with FEV1/FVC (forced vital capacity) < 0.70, the standard labels: (1) STAGE 1 – mild: FEV1≥ 80%; (2) STAGE 2 – moderate: 50%≤ FEV1 < 80%; (3) STAGE 3 – severe: 30%≤ FEV1 <50%; (4) STAGE 4 – very severe: FEV1 < 30%. Additionally, in this paper, the STAGE 0 label is assigned to patients without COPD (i.e., FEV1/FVC⩾0.70).

### Early COPD Stages

Nowadays, there have been many debates in the literature regarding the early stage of COPD or the so‐called asymptomatic COPD. Patients with COPD often underestimate the severity of the disease—primarily early morning and nighttime symptoms. The reasons may be the slow onset of their symptoms, cough due to a long cigarette smoking history, and dyspnea attributed to getting older. The majority of patients from a European cohort stated that they were not wholly frank with their doctors during visits when reporting their symptoms and quality of life.^[^ ^79^ ^]^

Around 36% of patients who describe their symptoms as mild‐to‐moderate also admit to being too breathless to leave the house. For these reasons, there are two validated questionnaires (i.e., CAT and Modified Medical Research Council (mMRC)) that allow clinicians to accurately and objectively assess COPD symptoms. CAT is a globally used, 8 question, patient‐filled questionnaire to evaluate the impact of COPD (cough, sputum, dyspnea, chest tightness) on health status. The range of CAT scores is 0–40. Higher scores denote a more severe impact of COPD on a patient's life.^[^ ^80^ ^]^ The mMRC Dyspnea Scale stratifies dyspnea severity in respiratory diseases, particularly COPD; it provides a baseline assessment of functional impairment attributable to dyspnea in respiratory diseases. Moreover, despite being highly symptomatic (mMRC⩾2 and CAT⩾10) and having at least one exacerbation, many COPD patients did not seek medical help, as they felt COPD symptoms as part of their daily smoking routine or due to aging. COPD awareness is poor among smokers; the smoker population underestimates their respiratory symptoms, while their exercise activity is reduced many times. Not surprisingly, 14.5% of the newly diagnosed COPD population was reported as asymptomatic in primary care clinics.^[^ ^81^ ^]^ Also, there is a high prevalence of COPD among smokers with no symptoms.^[^ ^82^ ^]^ Subjectively reported or observed clinical symptoms were not considered; instead, the analysis is based only on objectively measured parameters (i.e., physiological signals).

Spirometry as a screening tool for the early stage of the disease is not entirely robust.^[^ ^83^ ^]^ Indeed, spirometry can diagnose asymptomatic COPD, but its use is only recommended in smokers or individuals with a history of exposure to other noxious stimuli.^[^ ^84^ ^]^ Despite having an apparent normal lung function, smokers with normal spirometry but a low diffusing capacity of the lung for carbon monoxide (DLCO) are at significant risk of developing COPD with obstruction to airflow^[^ ^85^ ^]^—a category that may also be asymptomatic COPD. Moreover, no other disease markers are known to date to predict which patients with COPD of recent onset will progress to more significant disease severity.

Nonetheless, undiagnosed asymptomatic COPD has an increased risk of exacerbations and pneumonia. For these reasons, better initiatives are needed for the early diagnosis and treatment of COPD.^[^ ^86^ ^]^ Our method also aims at addressing the problem of early detection because it has an excellent accuracy at detecting early stages 1 and 2, which can also be detected with Spirometry. However, if our method can identify asymptomatic COPD that spirometry‐based methods cannot see remains an open question; to that end, a longitudinal study starting with a significant cohort is needed, which tracks the evolution of individuals over time to see if those predicted as asymptomatic COPD indeed develop the symptomatic form of the disease after several years.

## Conflict of Interest

The authors declare no conflict of interest.

## Author Contributions

M.U., L.U., P.B., and S.M. contributed to proposing the idea. C.Y., G.G., and A.L. contributed to analyze the fractional‐order dynamic characteristics of physiological signals. C.Y. and A.L. designed the DNN model and performed the experiments. M.U., L.U., and S.M. collected experimental data. C.Y., P.B., and M.U. contributed to write the manuscript. All authors provided feedback on the manuscript.

### Code and Data Availability

The code for reproducing the results is provided on: https://github.com/chenzhoy/Fractional‐dynamics‐foster‐deep‐learning‐of‐COPDstage‐prediction.

## Supporting information

Supporting Information

## Data Availability

The data that support the findings of this study are available from the corresponding author upon reasonable request.
