# Supplementary material for: Fractional Dynamics Foster Deep Learning of COPD Stage Prediction
Source: Adv Sci (Weinh). 2023 Feb 19;10(12):2203485. doi: 10.1002/advs.202203485 (PMC10131808; doi:10.1002/advs.202203485)
Supplement: Supplementary file 1 — Supporting Information [file ADVS-10-2203485-s001.pdf]

ADVANCED SCIENCE
Open Access

Supporting Information

for *Adv. Sci.*, DOI 10.1002/advs.202203485

Fractional Dynamics Foster Deep Learning of COPD Stage Prediction

Chenzhong Yin, Mihai Udrescu, Gaurav Gupta, Mingxi Cheng, Andrei Lihu, Lucretia Udrescu, Paul Bogdan*, David M. Mannino and Stefan Mihaicuta*

Fractional dynamics foster deep learning of COPD stage prediction

Chenzhong Yin[1], Mihai Udrescu[2,3*], Gaurav Gupta[1], Andrei Lihu[3], Lucretia Udrescu[4], Paul Bogdan[1*], David M Mannino[5], and Stefan Mihaicuta[6]

[1]Ming Hsieh Department of Electrical and Computer Engineering, University of Southern California, Los Angeles, CA, USA
[2]Department of Computer and Information Technology, Politehnica University, Timişoara, Romania
[3]Timişoara Institute of Complex Systems, Timişoara, Romania
[4]Department I – Drug Analysis, “Victor Babeş” University of Medicine and Pharmacy Timişoara, 2 Eftimie Murgu Sq., 300041 Timişoara, Romania
[5]University of Kentucky, College of Medicine, Lexington, KY, USA
[6]Department of Pulmonology, “Victor Babeş” University of Medicine and Pharmacy Timişoara, 2 Eftimie Murgu Sq., 300041 Timişoara, Romania
[*]Correspondence and requests for materials should be addressed to M.U. (email: mudrescu@cs.upt.ro) and P.B. (email: pbogdan@usc.edu)

ABSTRACT

Chronic obstructive pulmonary disease (COPD) is one of the leading causes of death worldwide, usually associated with smoking and environmental occupational exposures. Prior studies have shown that current COPD diagnosis (i.e., spirometry test) can be unreliable because the test can be difficult to do and depends on an adequate effort from the testee and supervision of the testor. Moreover, the extensive early detection and diagnosis of COPD is challenging. We address the COPD detection problem by constructing two novel COPD physiological signals datasets (4432 medical records from 54 patients in the WestRo COPD dataset and 13824 medical records from 534 patients in the WestRo Porti COPD dataset), demonstrating their complex coupled fractal dynamical characteristics, and performing a rigorous fractional-order dynamics deep learning analysis to diagnose COPD with high accuracy. We find that the fractional-order dynamical modeling can extract distinguishing signatures from the physiological signals across patients with all COPD stages—from stage 0 (healthy) to stage 4 (very severe). We exploit these fractional signatures to develop and train a deep neural network that predicts the suspected patients' COPD stages based on the input features (such as thorax breathing effort, respiratory rate, or oxygen saturation levels). We show that our COPD diagnostics method (fractional dynamic deep learning model) achieves a high prediction accuracy (98.66% $\pm$ 0.45%) on WestRo COPD dataset and can serve as an excellent and robust alternative to traditional spirometry-based medical diagnosis. Our fractional dynamic deep learning model (FDDLM) for COPD diagnosis also presents high prediction accuracy when validated by a dataset with different physiological signals recorded (i.e., 94.01% $\pm$ 0.61% for predicting the COPD stages in the WestRo COPD dataset with the model trained on the WestRo Porti COPD dataset, and 90.13% $\pm$ 0.89% for predicting in the WestRo Porti COPD with the model trained on WestRo COPD).

Supplementary information

Sensitivity, specificity, and precision rate of the confusion matrices

The WestRo COPD dataset consists of physiological signals recorded over consecutive patients from four Pulmonology Clinics in Western Romania (Victor Babeş Hospital – VB, Medicover 1 – MD1, Medicover 2 – MD2, and Cardio Prevent – CP clinics). This supplementary material displays detailed results about the confusion matrices presented in the manuscript (Figure 7). We generated the confusion matrices with our fractional dynamics deep learning model (FDDLM), the vanilla DNN model, and the LSTM model and by holding out data gathered at each institution—at a time—as test sets. (The vanilla DNN and LSTM models have a similar network structure with the fractional dynamics deep learning model, except the input size.) We analyze the sensitivity, specificity, and precision of these confusion matrices to measure the performance of each machine learning model. We present the results in Tables S1, S2, and S3. The results in these tables show that, despite LSTM and vanilla DNN models having high specificity, the prediction sensitivity and precision of these two models are not good (with the worst sensitivity values dropping to 36.77% and 35.33%). In contrast, our fractional dynamics deep learning model shows relatively high values across the three performance measurements. We conclude that our model predicts patients' COPD stage with higher accuracy than vanilla DNN and LSTM models—without overfitting—therefore representing an effective alternative

Medical institution	Parameters	Stage 0	Stage 1	Stage 2	Stage 3	Stage 4
	Sensitivity	97.96%	86.45%	99.99%	99.62%	99.99%
VB	specificity	99.99%	99.99%	97.01%	99.88%	99.79%
	Precision	99.99%	99.99%	94.42%	99.62%	98.84%
	Sensitivity	99.99%	98.76%	94.77%	97.12%	95.86%
MD1	specificity	98.85%	98.10%	99.71%	99.33%	99.99%
	Precision	93.29%	89.89%	99.52%	97.12%	99.99%
	Sensitivity	98.73%	98.20%	86.31%	94.51%	99.34%
MD2	specificity	96.70%	96.00%	98.70%	99.77%	99.78%
	Precision	83.87%	82.00%	99.44%	98.85%	98.68%
	Sensitivity	95.60%	95.83%	99.14%	99.35%	99.52%
CP	specificity	99.99%	99.66%	99.10%	99.23%	99.74%
	Precision	99.99%	97.87%	98.29%	97.47%	98.56%

Table S1. The COPD stage prediction results with the fractional dynamics deep learning model by holding out data gathered from each institution at a time as the test set.

to the spirometry-based diagnostic.

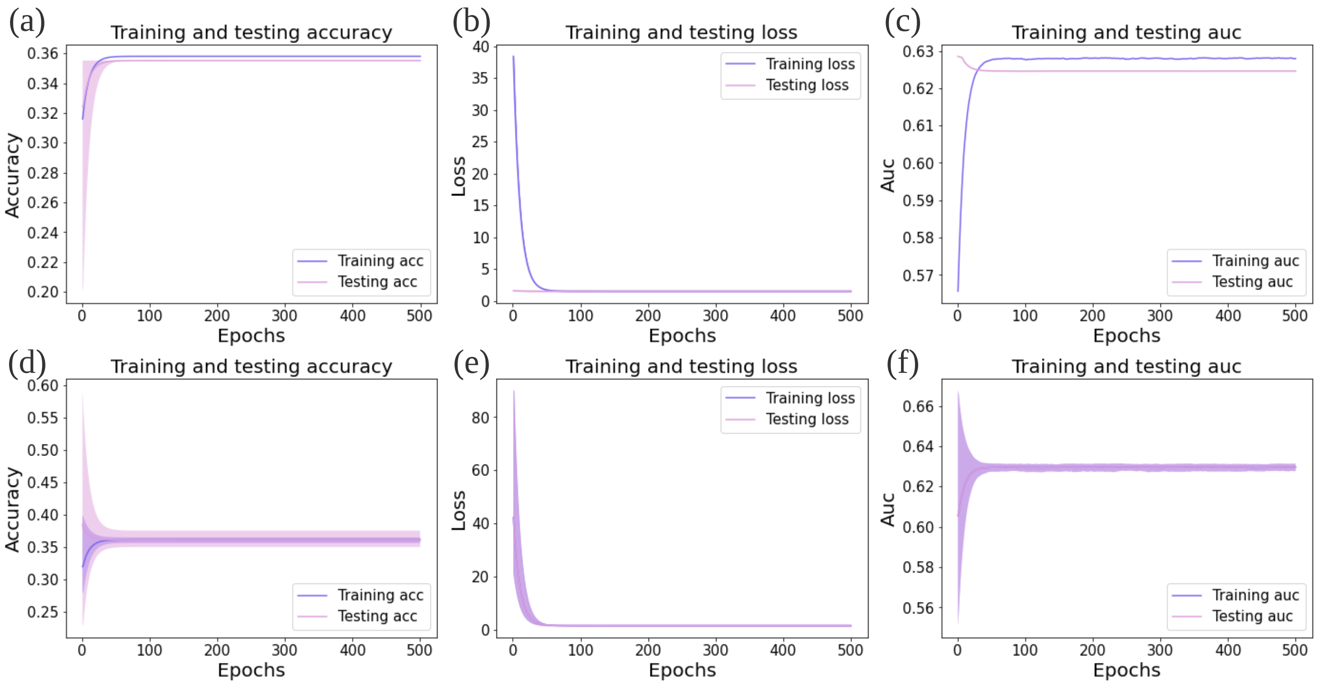

Figure S1. Training and testing results in terms of accuracy, loss, and AUC of the convolutional neural network model for the *k*-fold cross-validation and hold out validation. Training/testing accuracy (a), loss (b), and AUC (c) for convolutional neural network model under *k*-fold cross-validation, where the training processes utilize physiological signals. Training/testing accuracy (d), loss (e), and AUC (f) for convolutional neural network model under hold-out validation, where the training processes utilize physiological signals.

Convolutional neural network (CNN) training and testing results

In figure S1, panels (a), (b), and (c) illustrate the training and testing results in terms of accuracy, loss, and AUC for the

Medical institution	Parameters	Stage 0	Stage 1	Stage 2	Stage 3	Stage 4
VB	Sensitivity	91.16%	36.77%	94.09%	89.02%	74.71%
	specificity	94.90%	96.33%	93.34%	94.91%	96.91%
	Precision	73.22%	61.96%	87.72%	84.53%	81.41%
MD1	Sensitivity	79.61%	54.04%	86.62%	72.60%	77.24%
	specificity	96.75%	90.48%	93.54%	96.32%	94.07%
	Precision	79.60%	49.15%	89.88%	82.07%	66.27%
MD2	Sensitivity	97.25%	51.04%	92.67%	94.52%	81.64%
	specificity	96.59%	96.64%	93.93%	96.36%	98.52%
	Precision	81.57%	71.53%	88.84%	88.51%	68.93%
CP	Sensitivity	78.48%	52.09%	88.51%	97.80%	79.61%
	specificity	96.81%	91.01%	92.11%	96.61%	99.56%
	Precision	81.04%	51.78%	87.44%	85.57%	96.80%

Table S2. The COPD stage prediction results with the vanilla DNN model by holding out data gathered from each institution at a time as the test set.

Medical institution	Parameters	Stage 0	Stage 1	Stage 2	Stage 3	Stage 4
VB	Sensitivity	99.99%	50.63%	80.91%	87.12%	88.82%
	specificity	91.90%	92.55%	96.89%	99.99%	96.81%
	Precision	65.33%	52.98%	92.90%	99.99%	83.43%
MD1	Sensitivity	75.16%	73.29%	78.91%	73.07%	82.76%
	specificity	96.23%	86.16%	98.95%	97.22%	94.18%
	Precision	76.16%	47.39%	98.02%	85.87%	68.18%
MD2	Sensitivity	66.46%	35.33%	97.07%	70.72%	93.42%
	specificity	98.42%	93.19%	86.26%	98.98%	93.31%
	Precision	87.50%	47.97%	80.86%	94.57%	68.93%
CP	Sensitivity	99.99%	53.13%	77.80%	89.35%	99.03%
	specificity	92.32%	91.14%	99.99%	99.80%	97.13%
	Precision	66.91%	49.76%	99.99%	99.28%	86.13%

Table S3. The COPD stage prediction results with the LSTM model by holding out data gathered from each institution at a time as the test set.

CNN model trained with physiological signals (raw data) under k-fold cross-validation ($k = 5$). The testing accuracy of CNN indicates a lower predicting accuracy (36.12%±0.001%) compared with our fractional dynamics model (98.66%±0.447%). In figure S1, panels (d), (e), and (f) present the training and testing results in terms of accuracy, loss, and AUC for the CNN model trained with physiological signals (raw data) under hold-out validation. The testing accuracy under hold-out validations is 35.51%±0.009%, significantly lower than our model's predicting accuracy (95.88%±1.76%). Hence, we conclude that our fractional-dynamics deep learning model predicts patients' COPD stages with higher accuracy than the vanilla DNN, LSTM, and CNN models trained with physiological signals.

Hurst exponents of physiological signals

In this section, we show the Hurst exponents in non-derived signals (Thorax, Oxygen Saturation, Pulse, Plethysmograph, Nasal Pressure, and Abdomen) among COPD patients for the intermediate-stage patients ($q \in [-5,5]$). The Hurst exponent measures the long-term memory of time series; different Hurst exponent values reveal different evolving variations in time series with different fractal features. Figure S2 presents the $H(q)$ confidence intervals for Abdomen, Thorax, and Pulse signals, for different q values, across different COPD stages (i.e., 0, 1, 2, 3) with 95% confidence intervals; Figure S3 presents the $H(q)$ confidence intervals for Nasal Pressure, Oxygen Saturation, and Plethysmograph signals, for different q values, across different stages (0, 1, 2, 3) with 95% confidence intervals. In both Figure S2 and Figure S3, the $H(q)$ of physiological signals extracted from healthy people (stage 0) are plotted as purple patterns in each panel for reference. From these two figures, we notice that different stages have different $H(q)$ confidence intervals under the same q values, which is the evidence that all physiological signals extracted from patients with different COPD stages have different fractional dynamic characteristics (for reference, Figure S4 and Figure S5 present the comparison of terms of $H(q)$ intervals for COPD patients for different q values ($q \in \{-5,0,5\}$). Hence, it makes sense to analyze the spatial coupling between these physiological processes (signals) across time to investigate the different fractional features across signals recorded from different COPD patients.

Figure S6 to show the scaling functions of physiological signals extracted from all stage 4 patients and the healthy people (stage 0) in our dataset (where $q \in [-5,5]$); the purple areas are the confidence intervals. We also used the MF-DFA toolbox provided by[1] to recompute the multifractal scaling functions of the signals collected from stage 0 and 4 participants (we choose the same participants in Figure 2 for reanalyzing the $S(q)$). The results are shown in Figure S7

Standard deviation analysis and MSE analysis for scaling functions

To show the fractal difference between raw signals recorded from stage 0 and 4 participants in detail, we generated Figure S9 to show the correlations between scale (s) vs. standard deviations (STDs) of $S_F(q)$ and q values vs. STDs of $S_F(s)$. In Figure S9, Panels (a) and (b) respectively show the s vs. STDs of $S_F(q)$ for stage 0 and 4 participants across 6 physiological signals (abdomen, nasal pressure, oxygen saturation, plethysmograph, pulse, and thorax). When $s = L$, in stage 0, the STD for all six signals is close to zero; in stage 4, the STDs for all signals are larger than 0.5 (except nasal pressure). This observation is also similar to Fig. 1 in Mukli et al. 's work[1], where the multifractal signals have smaller STDs of $log(S(q,L))$ than the multifractal noise (monofractal). Accordingly, we can claim that the raw signals across Stage 0 and Stage 4 participants have different fractal features. To measure the error in terms of scale and fluctuation, we show the mean square error (MSE) of the regressed thin pink lines in Figure 2. We show the results in Figure S10, where (a) and (b) show the MSE for stage 0 and 4 participants, respectively.

Error analysis and learning process visualization

Although the fractional dynamics deep learning model FDDLM provides relatively high prediction accuracy under k-fold cross-validation and hold-out validation, the detection accuracy drops a bit in the hold-out validation (from 98.66% to 95.88%). The reason is that the data recorded from each medical institution are unbalanced, as our cohort is a real-life population. The Victor Babes (VB) and Cardio Prevent (CP) are two large clinics with COPD patients who are more willing to get the diagnosis or medical treatment in large units or hospitals than in small clinics—especially in the case of severe and very severe COPD stages. Thus, signals recorded from VB and CP are more comprehensive/diverse than from Medicover 1 (MD1) and Medicover 2 (MD2). In the hold-out section, we balanced the data across different institutions using over-sampling and under-sampling approaches. Indeed, the unbalanced data collection is the leading cause of the prediction accuracy drop in the hold-out section. In Figure S8, we present the data distribution across the medical institutions; in the left-hand side panel, we have the distribution of the absolute number of individuals, while in the right-hand side panel, we present the percentage of COPD stages for each institution. Both panels emphasize the data unbalance problem—MD1 and MD2 have a small number of individuals, and only CP has individuals representing all COPD stages. To handle these imbalanced data across different institutions, we perform over-sampling and under-sampling techniques to guarantee that all institutions have a similar number of testing samples across different COPD stages (e.g., since MD2 does not have stage 4 samples, we move two stage-4 patients from VB to MD2 and over-sample/under-sample the data to ensure the data balance).

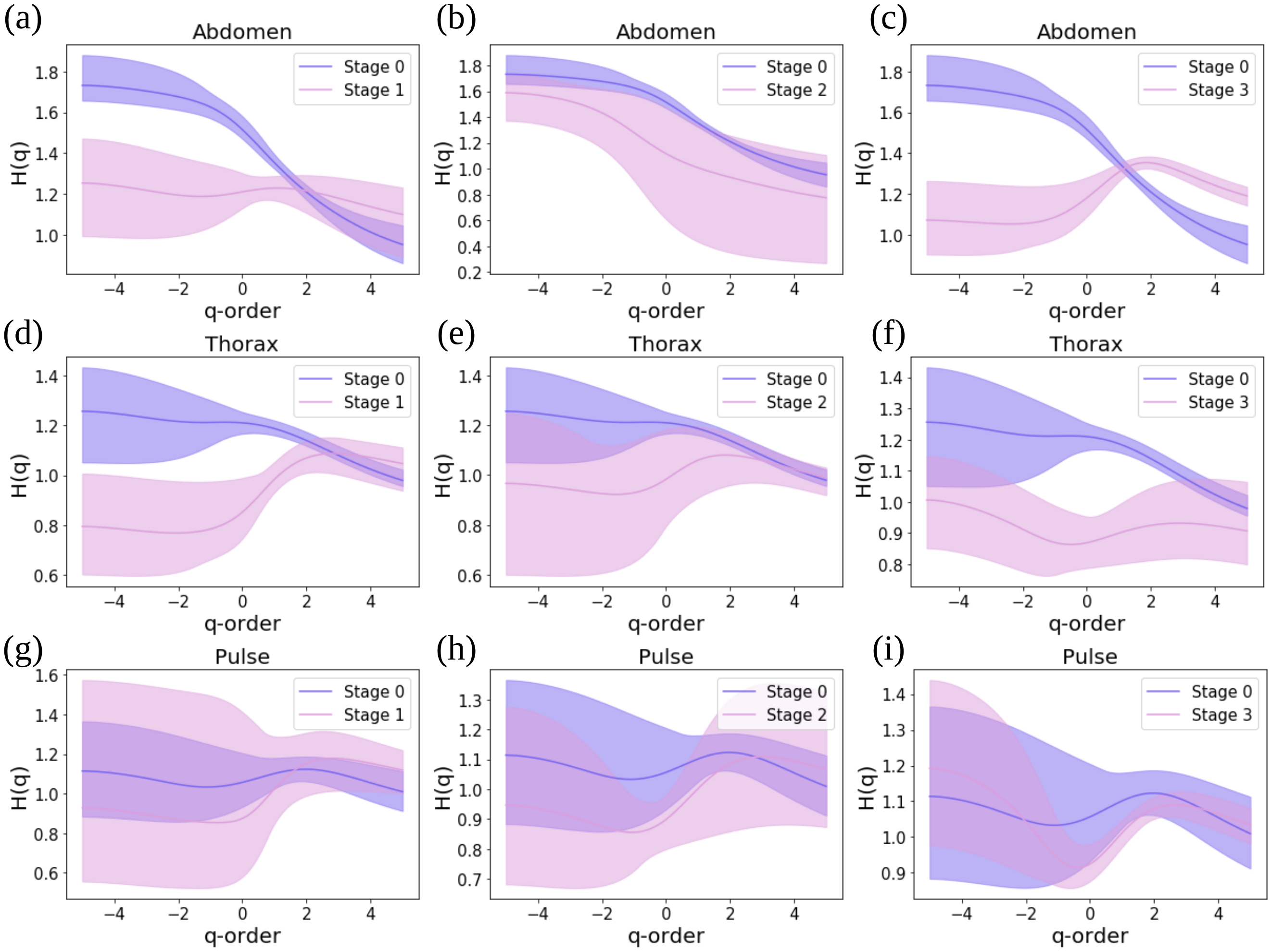

Figure S2. Multifractal analysis of 3 non-derived physiological signals (Abdomen, Thorax, and Pulse) extracted from non-COPD individuals (i.e., stage 0) and stage 4 COPD patients with 95% confidence interval. We present the generalized Hurst exponent ($H(q)$) as a function of q-th order moments ($q \in [-5,5]$) for Abdomen (a), Thorax (d), and Pulse (g) signals extracted from stage 1 patients; $H(q)$ as a function of q-th order moments ($q \in [-5,5]$) for Abdomen (b), Thorax (e), and Pulse (h) signals extracted from stage 2 patients; $H(q)$ as a function of q-th order moments ($q \in [-5,5]$) for Abdomen (c), Thorax (f), and Pulse (i) signals extracted from stage 3 patients. For reference, the $H(q)$ function for all physiological signals (Abdomen, Thorax, and Pulse) extracted from the healthy people (stage 0) are plotted in (a-i) panels as purple curves.

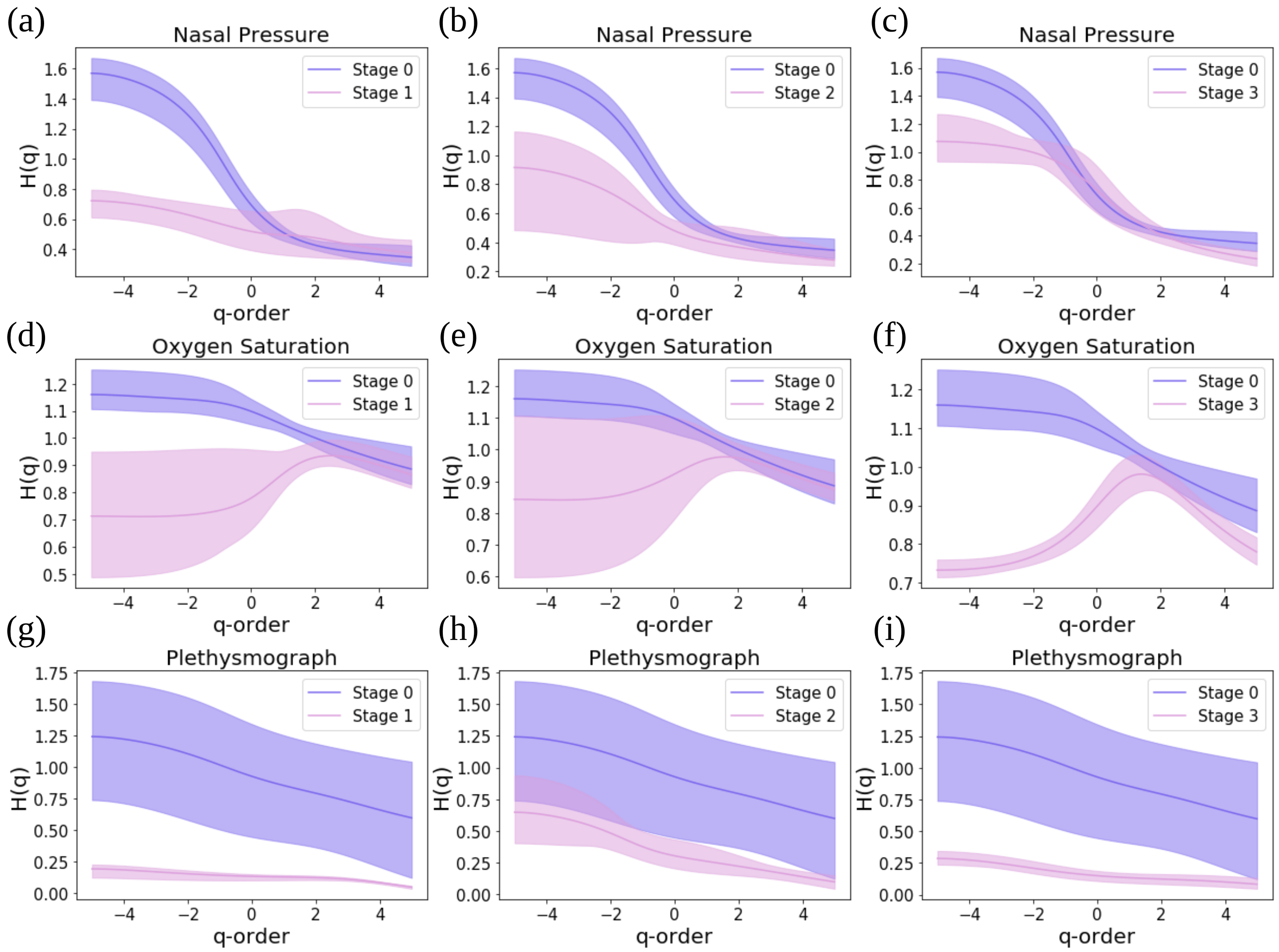

Figure S3. Multifractal analysis of 3 non-derived physiological signals (Nasal Pressure, Oxygen Saturation, and Plethysmograph) extracted from non-COPD individuals (i.e., stage 0) and stage 4 COPD patients with 95% confidence interval. We present the generalized Hurst exponent ($H(q)$) as a function of q-th order moments ($q \in [-5, 5]$) for Nasal Pressure (a), Oxygen Saturation (d), and Plethysmograph (g) signals extracted from stage 1 patients; $H(q)$ as a function of q-th order moments ($q \in [-5, 5]$) for Nasal Pressure (b), Oxygen Saturation (e), and Plethysmograph (h) signals extracted from stage 2 patients; $H(q)$ as a function of q-th order moments ($q \in [-5, 5]$) for Nasal Pressure (c), Oxygen Saturation (f), and Plethysmograph (i) signals extracted from stage 3 patients. For reference, the $H(q)$ function for all physiological signals (Nasal Pressure, Oxygen Saturation, and Plethysmograph) extracted from the healthy people (stage 0) are plotted in the (a-i) panels as purple curves.

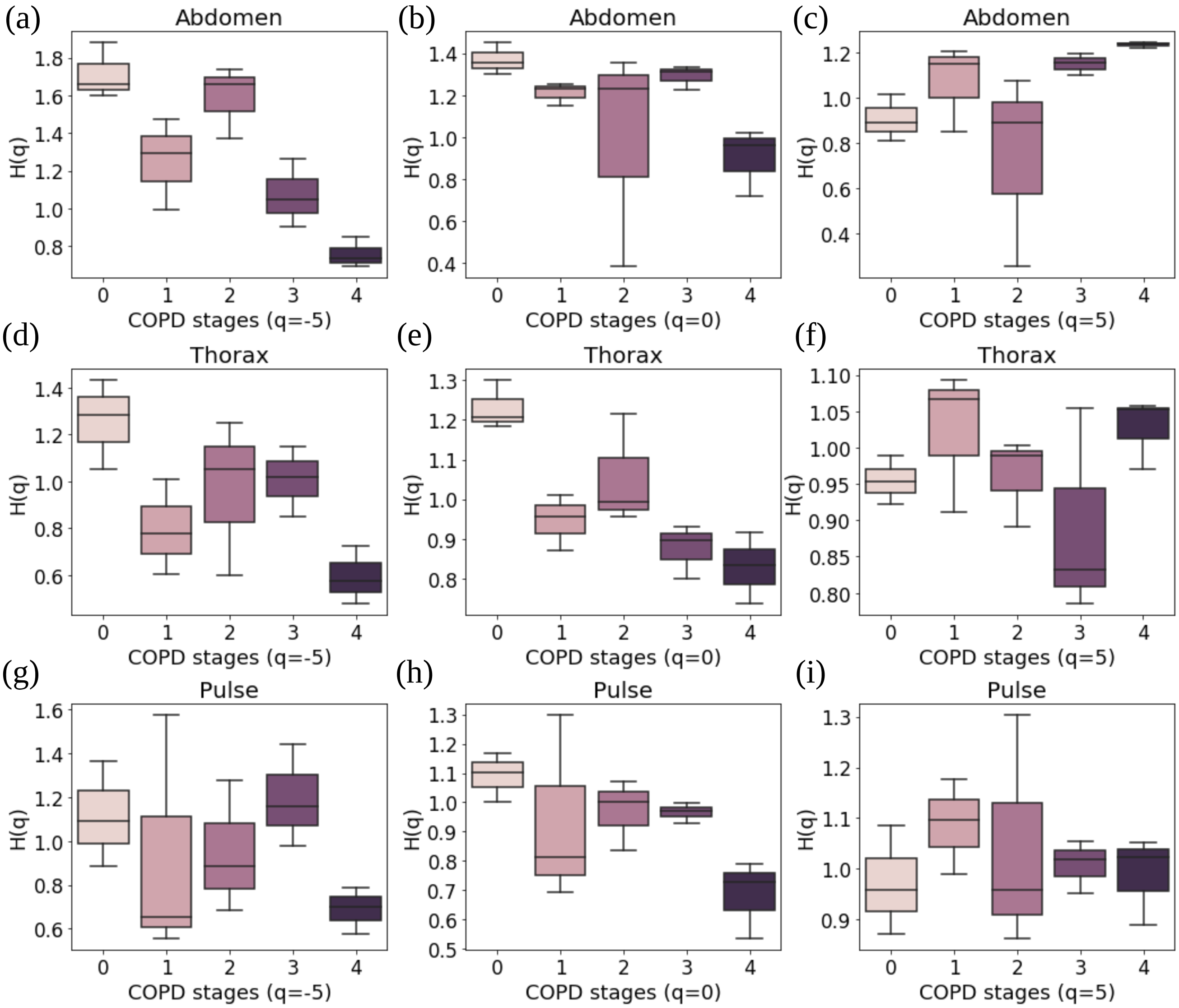

Figure S4. Comparison in multifractal features between 3 non-derived physiological signals extracted from patients with different COPD stages. We plot the generalized Hurst exponent ($H(q)$) for Abdomen signals extracted from COPD patients in different stages under different q-th order moments ($q = -5$ (a), $q = 0$ (b), and $q = 5$ (c)); Generalized Hurst exponent ($H(q)$) for Thorax signals extracted from COPD patients with different stages under different q-th order moments ($q = -5$ (d), $q = 0$ (e), and $q = 5$ (f)); $H(q)$ for Pulse signals extracted from COPD patients in different stages under different q-th order moments ($q = -5$ (g), $q = 0$ (h), and $q = 5$ (i)).

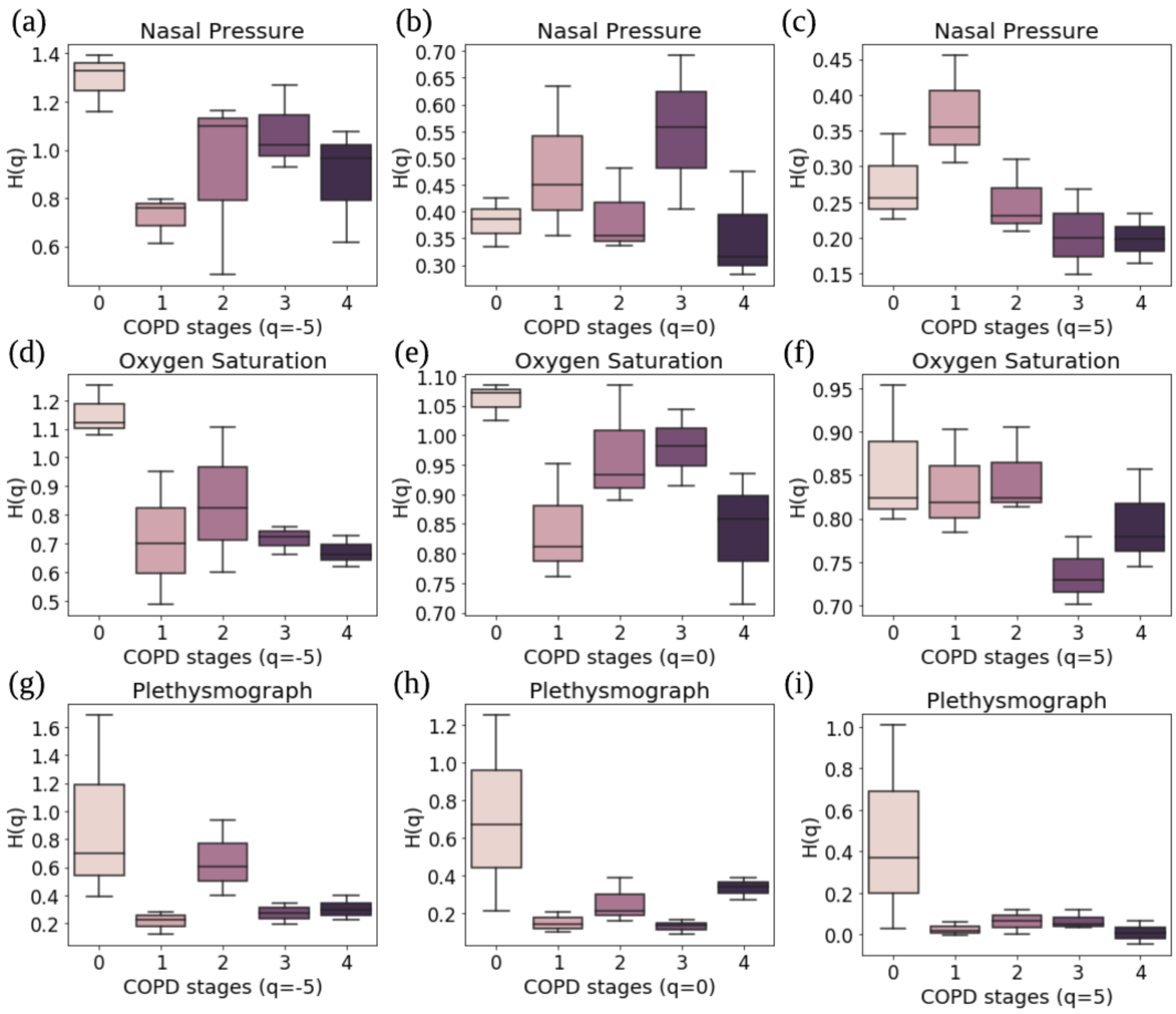

Figure S5. Comparison of multifractal features between 3 non-derived physiological signals extracted from patients with different COPD stages. We plot the generalized Hurst exponent ($H(q)$) for Nasal Pressure signals extracted from COPD patients in different stages under different q-th order moments ($q = -5$ (a), $q = 0$ (b), and $q = 5$ (c)); $H(q)$ for Oxygen Saturation signals extracted from COPD patients with different stages under different q-th order moments ($q = -5$ (d), $q = 0$ (e), and $q = 5$ (f)); $H(q)$ for Plethysmograph signals extracted from COPD patients in different stages under different q-th order moments ($q = -5$ (g), $q = 0$ (h), and $q = 5$ (i)).

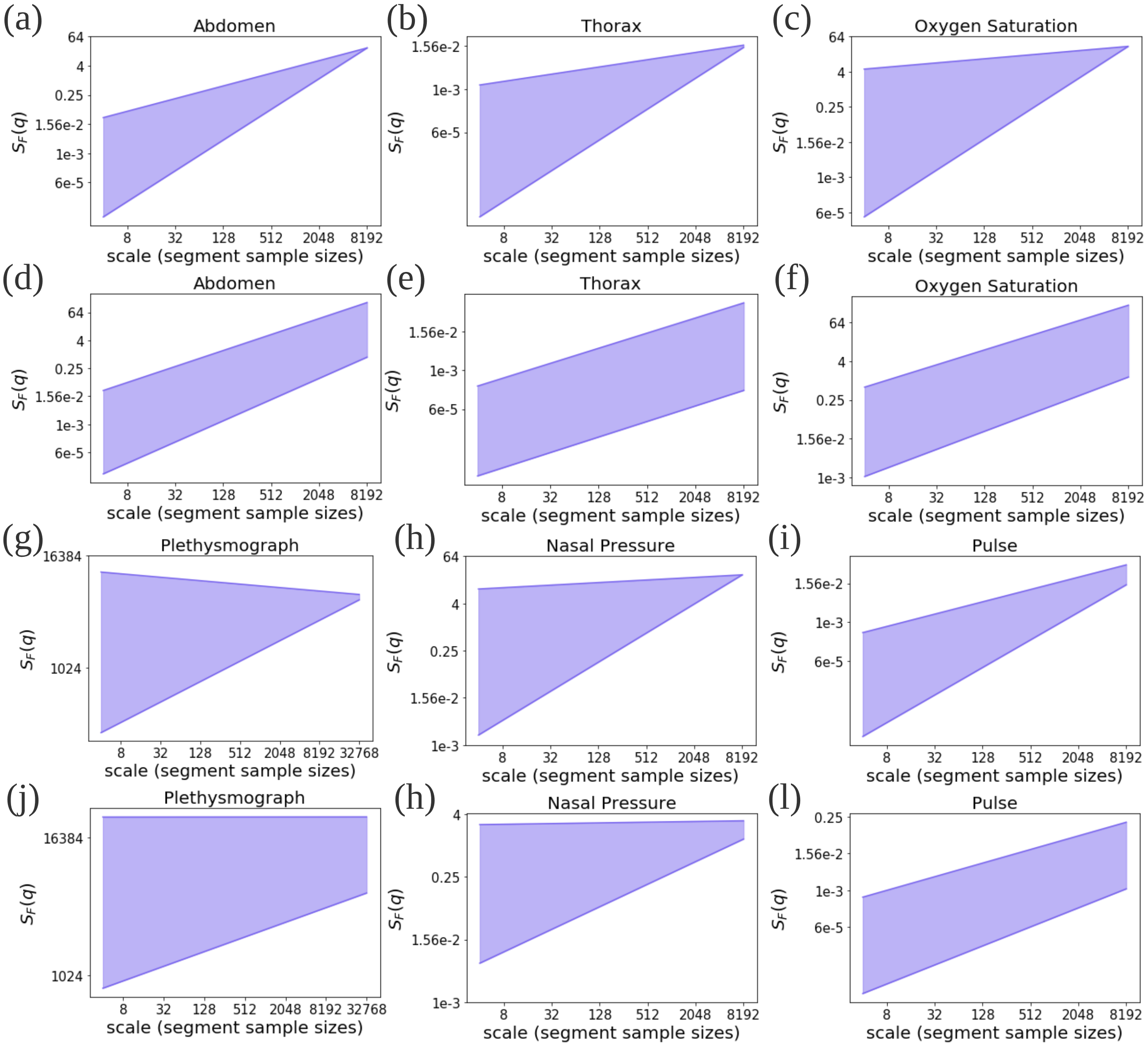

Figure S6. The geometry of fluctuation profiles for the COPD-relevant physiological signals recorded from healthy people and stage 4 patients. We compute the scaling function from non-derived physiological signals (Abdomen, Thorax, Oxygen Saturation, Plethysmograph, Nasal Pressure, and Pulse) with the exponents $q \in [-5, 5]$. Panels (a-c) and (g-i) correspond to all healthy people in our datasets (stage 0), while (d-f) and (j-l) correspond to all the stage 4 COPD patients in our datasets.

To provide further insight, we present the learning process in the hidden layers of our fractional dynamics deep learning model for *k*-fold and hold-out validation by employing the t-Distributed Stochastic Neighbor Embedding (t-SNE) visualization algorithm. Besides the input-, dropout-, and output-layers, we have two hidden dense layers in our deep learning model (the first layer has 300 neurons and the second layer has 100 neurons). The t-SNE technique is an approach to reduce data dimensionality in two or three-dimension maps. In this work, we regard the outputs of the first dense layer as a 300-dimension coordinate and the second dense layer as a 100-dimension coordinate. Then, we employ the t-SNE technology to reduce these coordinates to two-dimension coordinates and visualize the learning processes in these two hidden dense layers. Figure S11 presents the learning processes visualization of the first hidden layer. In Figure S11, panels (a-d) present the t-SNE results for hold-out validation, and panels (e-i) show the t-SNE results for *k*-fold cross-validation. The color of scatters presents the label of each individual case, and each point's shape indicates the prediction results of our fractional dynamics deep learning model (where the round markers indicate the correct prediction results and the 'x' markers indicate errors). Figure S11 (a-d)

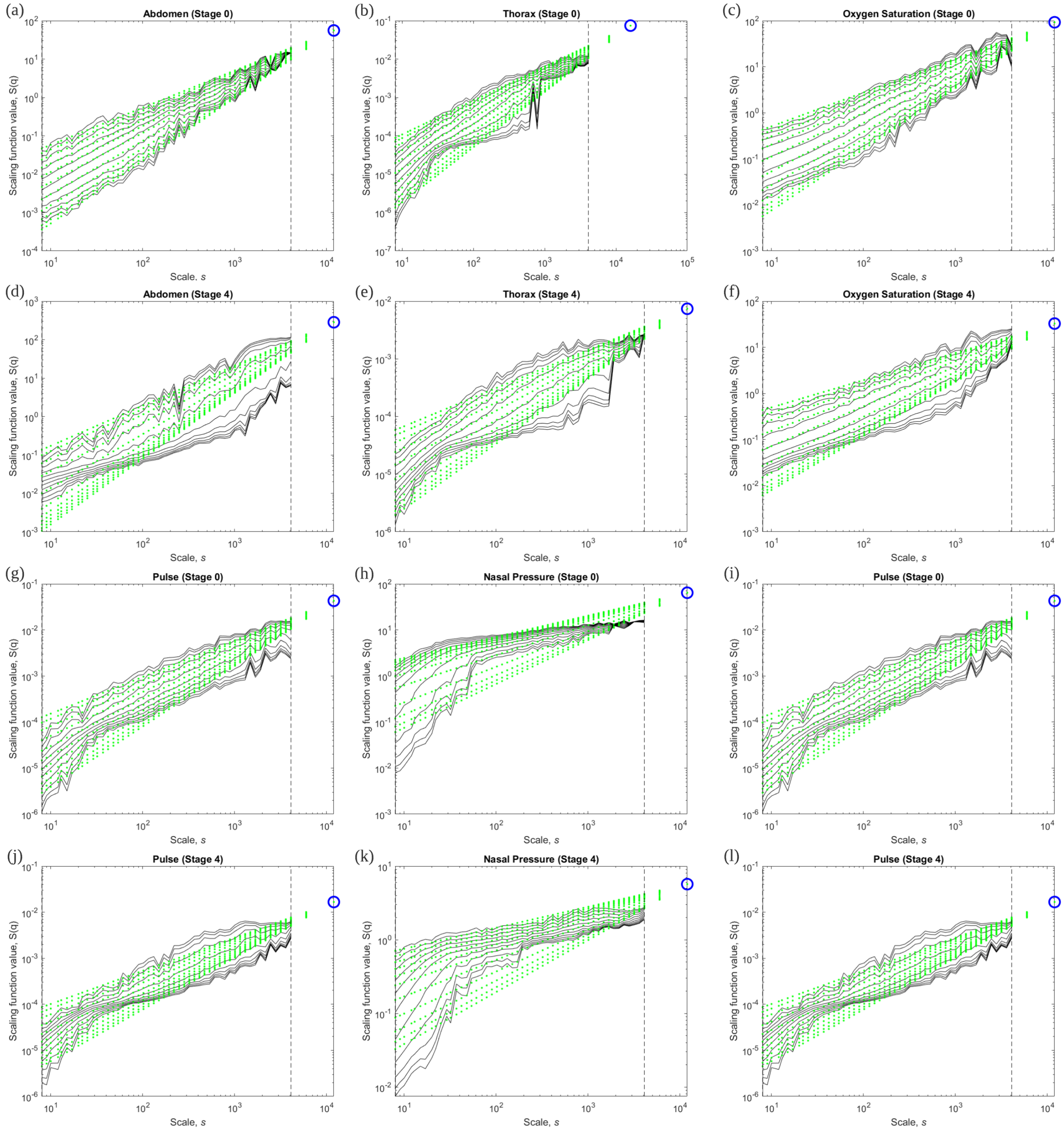

Figure S7. The geometry of fluctuation profiles for the COPD-relevant physiological signals recorded from a normal abdomen and a stage 4 COPD abdomen. We use the toolbox provided by [1] to calculate the scaling functions from 6 raw physiological signals: Abdomen, Thorax, Oxygen Saturation, Plethysmograph, Nasal Pressure, and Pulse, where the exponents are $q \in$ [-5, 5]. Panels (a-c) and (g-i) with signals recorded from a healthy person, (d-f), and (j-l) with signals recorded from a representative stage 4 COPD patient.

hold out the data recorded from different institutions as test sets (VB (a), MD1 (b), MD2 (c), and CP (d)), and (e-i) hold out the 0-20% (e), 20%-40% (f), 40%-60% (g), 60%-80% (h), 80%-100% (i) data as test sets, respectively. In Figure S11, we can find that scatters under the same category tend to build clusters, and some clusters overlap. The overlapping patterns are difficult to learn for the deep learning model (errors mostly occur in these overlapping ranges), and the isolated clusters with clear boundaries are the easiest part to recognize for the deep learning model. By comparing the t-SNE figures across hold-out

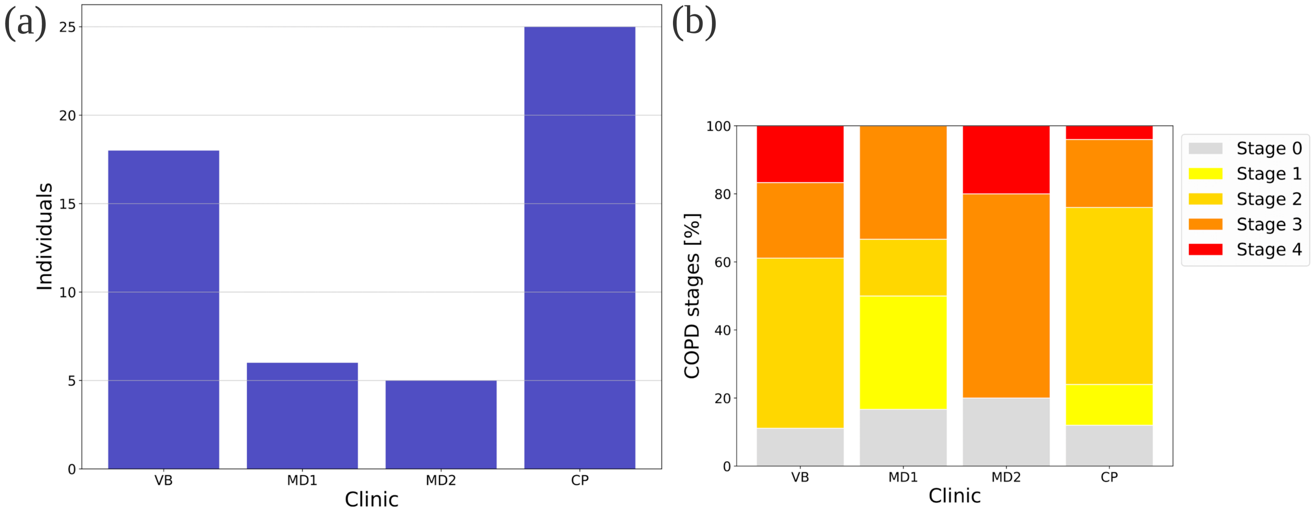

Figure S8. (a). Data distribution across the medical institutions (b). Percentage of COPD stages for each medical institution

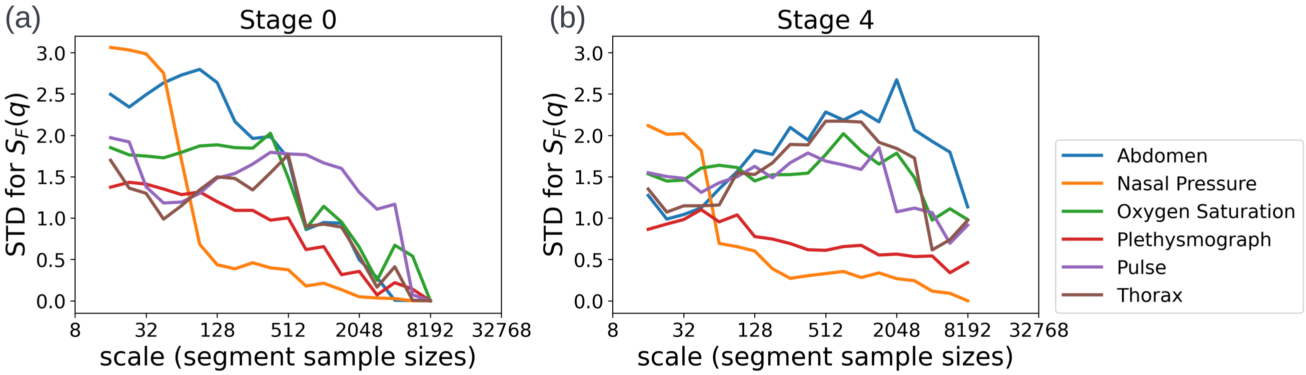

Figure S9. Scale (s) vs. standard deviation (STD) of $S_F(q)$ between stage 0 (a) and stage 4 (b) participants.

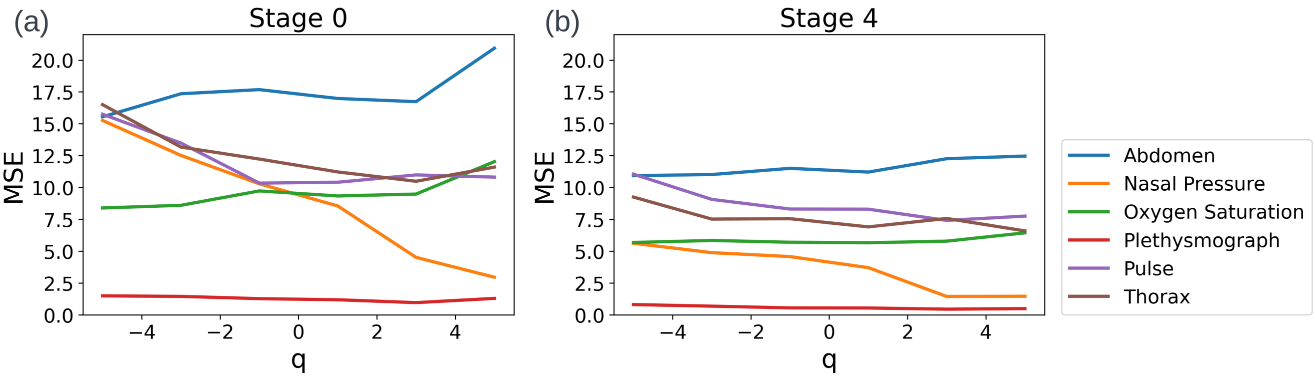

Figure S10. Mean squared error (MSE) for the regressed lines in Figure 2 for stage 0 and stage 4 participants.

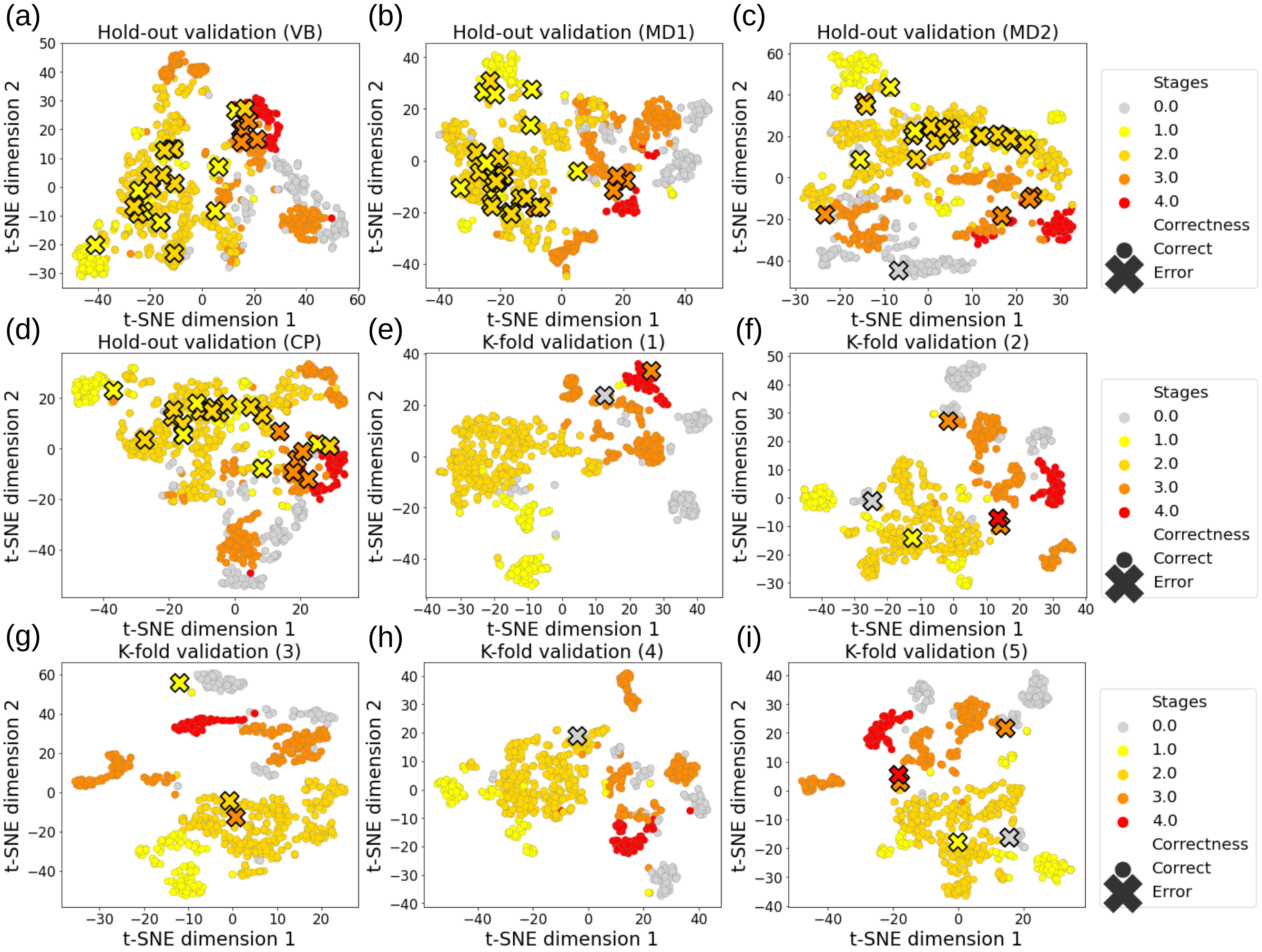

Figure S11. Representation of the learning process about hold-out and *k*-fold cross-validation in the first hidden layer. We present the t-SNE visualization of the first hidden layer in the fractional dynamics deep learning model of the COPD dataset under hold-out (a-d) and *k*-fold (e-i) cross-validation. In each scatterplot, the colors of the points indicate the classes of the corresponding COPD stages, and the shape of the points represents the fractional dynamics deep learning model's prediction results (i.e., the round shape indicates a correct prediction, and the 'x' mark indicates an error).

validation (Figure S11 (a-d)) and *k*-fold cross-validation (Figure S11 (e-i)), we find that the scatters in hold-out validation have more extensive overlapping ranges than the scatters in *k*-fold cross-validation. This observation illustrates that the datasets used in hold-out validation are harder to distinguish for the deep learning model than the dataset used in *k*-fold cross-validation.

Figure S12 presents the learning processes visualization of the last hidden layer in our fractional dynamics deep learning model. Figure S12 (a-d) hold out the data gathered from different institutions as test sets (VB (a), MD1 (b), MD2 (c), and CP (d)), and (e-i) hold out the 0-20% (e), 20%-40% (f), 40%-60% (g), 60%-80% (h), 80%-100% (i) data as test sets, respectively. As one can observe, Figure S12 has the same occurrence as in Figure S11; namely, that the datasets used in hold-out validation are more difficult to learn than the dataset used in *k*-fold cross-validation. Moreover, by comparing Figure S11 and Figure S12, we observe that overlapping patterns in each Figure S12 panel are narrower than the corresponding panel in Figure S11. This provides numerical evidence that our fractional dynamics deep learning model FDDLM provides a feasible learning process and gives desired prediction results.

Linear classifier of coupling matrices

Besides the deep learning architecture in our fractional dynamics deep learning model FDDLM, we also utilize linear classifier models to mine the complexity of coupling matrices A. Logistic regression is traditionally a linear classifier; it is one of the most used two-class (and multi-class) classification machine learning algorithms[2]. When we use logistic regression instead of deep

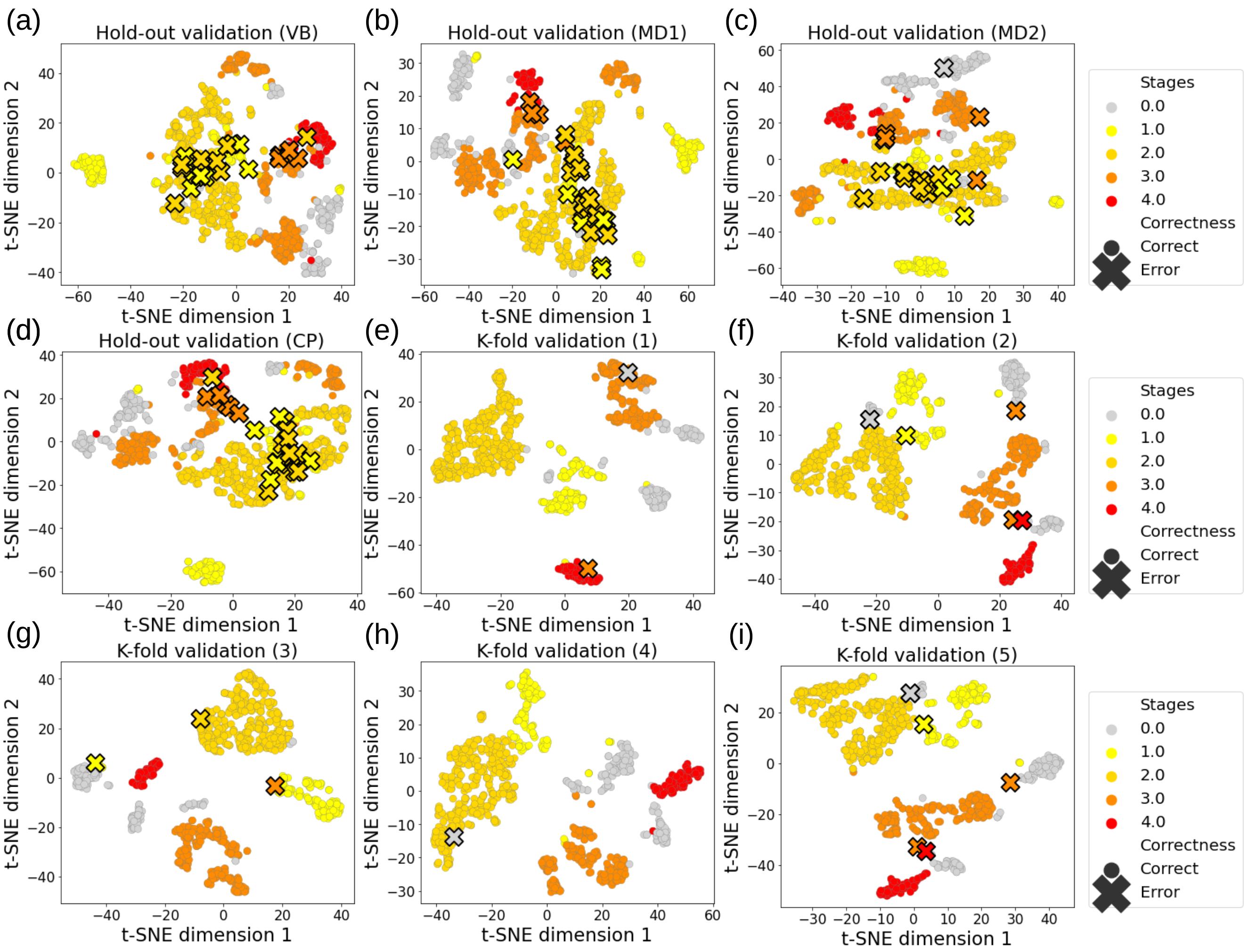

Figure S12. Representation of the learning process about hold-out and *k*-fold cross-validation in the last hidden layer. We present the t-SNE visualization of the last hidden layer in the fractional dynamics deep learning model of the COPD dataset under hold-out (a-d) and *k*-fold (e-i) cross-validation. In each scatterplot, the colors of the points indicate the classes of the corresponding COPD stages, and the shape of the points represents the fractional dynamics deep learning model's prediction results (i.e., the round shape indicates a correct prediction, and the 'x' mark indicates an error).

learning on the A matrices, the accuracy of COPD prediction is $94.61\% \pm 0.98\%$ with k-fold and $86.68\% \pm 0.82\%$ with hold-out validation. The COPD prediction accuracy for logistic regression is lower with the k-fold validation and significantly lower with the hold-out validation than the fractional dynamics deep learning model (i.e., $98.66\% \pm 0.45\%$ and $95.88\% \pm 1.76\%$, respectively). We show the confusion matrices for logistic regression in Figure S13; panels (a-e) are the prediction results on test sets for the k-fold validation, and (f-i) panels show the prediction results for the hold-out validation. Of note, for the linear classifier model, the accuracy for the k-fold validation is significantly higher than the accuracy for hold-out validation. The explanation is that our data is unbalanced in different medical institutions (for detailed information, see the *Supplementary material*'s section *Error analysis and learning process visualization*).

Besides the logistic regression, we also investigate another classifier model as a reference, namely, SVM. Support-vector machines (SVM) are a particular case of linear classifiers based on the margin maximization principle[3]. When using SVMs instead of deep learning on the A matrices, the accuracy of COPD prediction becomes $94.74\% \pm 0.24\%$ with k-fold and $92.62\% \pm 0.39\%$ with hold-out validation. We show the confusion matrices for SVM under k-fold and hold-out validations in Figure S14. In Figure S14, panels (a-e) are the prediction results on test sets for the k-fold validation, and (f-i) panels show the prediction results for the hold-out validation. Like the logistic regression model, the k-fold validation accuracy is significantly higher than the accuracy for hold-out validation in the SVM model. The reason is that our data is unbalanced across the institutions. We conclude that our fractional dynamics deep learning model outperforms these two linear classifier models (i.e., logistic regression and SVM) in terms of prediction accuracy under k-fold and hold-out validation. Indeed, learning from many matrices A is a complicated process that requires deep learning.

Standard questionnaires, exacerbation history, and comorbidities of COPD patients

In this section, we provide the data description about the standard questionnaires (CAT and MRC), exacerbation history, and comorbidities about all the COPD patients in our datasets. Questionnaires are recommended for the management of COPD. Since 2011, the GOLD guidelines have included the following questionnaires in the assessment of COPD patients: the modified Medical Research Council (mMRC) dyspnea scale[4], the COPD assessment test (CAT)[5], and the clinical COPD questionnaire (CCQ); they are carefully-designed high-quality questionnaires, but information on the feasibility for routine use is scarce. Nonetheless, questionnaires are both quick to complete and have good acceptability by the patient. In addition, the agreement between electronic and paper versions of the questionnaires was high. Figure S15 presents the CAT and MRC scores for COPD patients across different stages in our paper's WestRo cohort. However, we made sure that our diagnoses—that lead to classifying patients in COPD stages 0 to 4—are reliable by reviewing each case after several months (consisting of a complete medical check-up, including spirometry).

We also collected data about the history of exacerbation for all the patients. When they were evaluated, their condition was stable. The data about the exacerbation history in the previous year for our patients is 16 patients without exacerbations, 25 patients with one exacerbation, 5 patients with two exacerbations, and 1 with three exacerbations. We considered it essential to make all the measurements and evaluations in a stable COPD phase of the disease. Therefore, we performed all functional tests when patients were not experiencing an acute exacerbation. The reason is that there is an increase in hyperinflation and gas trapping during an exacerbation, with a reduced expiratory flow and increased dyspnea[6].

Exacerbations are important in the management of COPD patients. There is an *frequent exacerbator* phenotype with an increased risk of hospitalization and death[7]. Exacerbations of COPD have a considerable impact on patients' health status and exercise capacity and have a cumulative effect on lung function[7]. However, longitudinal changes in FEV1 are not significantly associated with the exacerbation risk. Exacerbations can be found in any COPD stage[8]. In addition, a single COPD exacerbation may also result in a significant increase in lung function decline rate[9]. To investigate the impact of exacerbations, we pick 4 patients from each COPD stage (i.e., stage 2, 3, and 4) and hold out untrained signal samples as test sets (where 2 of them have 2 or more exacerbations and the other 2 have less than 2 exacerbations). We apply our model to make predictions about these test sets to reveal whether the exacerbation history will influence our prediction accuracy (the reason for choosing 4 patients in each stage from our datasets is that we want to maintain the test sets balanced during the prediction process). In stage 2, the prediction accuracy of samples gathered from patients with less than 2 exacerbations (category 1) is 99.01% and the prediction accuracy of samples generated from patients with more than 2 exacerbations (category 2) is 98.07%. In stage 3, the prediction accuracy is 99.28% for category 1 and 98.23% for category 2. In stage 4, the prediction accuracy of category 1 is 97.88%, and of category 2 is 98.22%. Hence, we conclude that the distribution of exacerbations history and comorbidities across patients and stages correlated with the high prediction rate of our method suggests that our fractal dynamics deep learning model FDDLM is not influenced by comorbidities or exacerbation history. (We have patients with exacerbation history in all COPD stages, except—of course—stage 0 COPD; we also have many COPD stages represented in each comorbidity, please see Figure S16.)

Comorbidities in COPD are expected at any stage of the disease [10]. The most common comorbidities accompanying COPD include cardiovascular diseases, metabolic disorders, osteoporosis, musculo-skeletal dysfunction, anxiety/depression, cognitive impairment, gastrointestinal diseases, and respiratory conditions such as asthma, bronchiectasis, pulmonary fibrosis, and lung

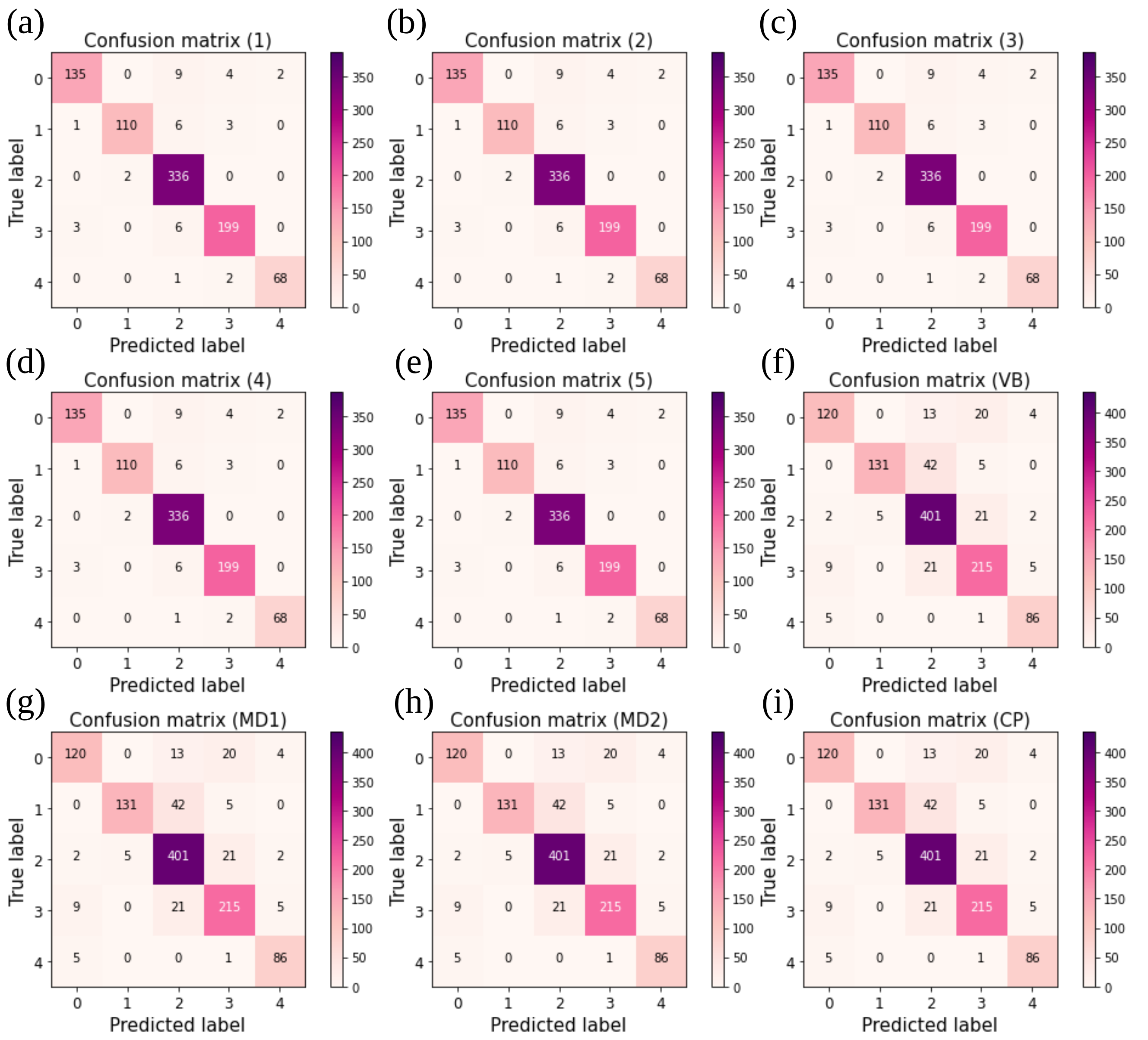

Figure S13. The confusion matrices resulted from the logistic regression model. The k-fold cross validation (k=5): in each iteration, we hold out 0-20% (a), 20%-40% (b), 40%-60% (c), 60%-80% (d), and 80%-100% data (e) as test sets. For the hold-out validation, we built the test sets by holding out data recorded from one institution at a time: VB (f), MD1 (g), MD2 (h), and CP (i).

cancer[11]. Comorbidities are known to pose a challenge in the assessment and effective management of COPD. However, the mechanistic links between COPD and its comorbidities are still not fully understood. The variability of the clinical presentation in COPD interacts with comorbidities to form a complex clinical scenario for clinicians to deal with[12]. As a result, attention needs to be paid to assessing and managing comorbidities in COPD in both clinical and research settings. In addition, for the effective management of comorbidities in COPD, there is a need for reliable measurement tools that can assist in improving clinical outcomes. In this work, we record the following comorbidities: Cardiovascular comorbidities (CC); Cancers (CA); Metabolic comorbidities (MC); Psychiatric comorbidities (PC); and Renal disease (RD). The results are shown in Figure S16 (b).

Convergence of coupling matrix

To evaluate our fractional dynamics deep learning model's efficiency, we investigate the sufficient length of the signals to make

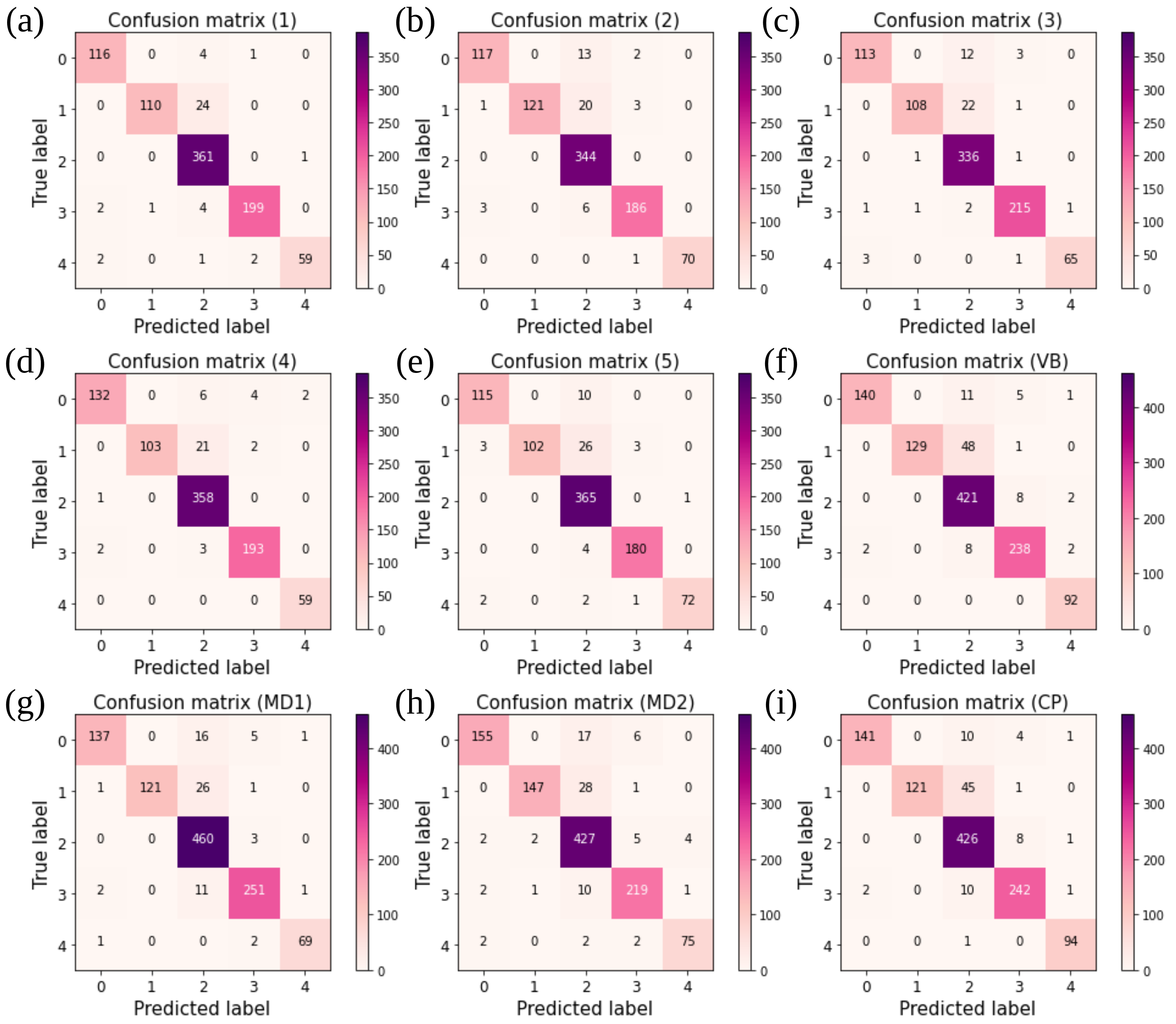

Figure S14. The confusion matrices resulted for the SVM. For the k-fold cross validation (k=5): in each iteration, we hold out 0-20% (a), 20%-40% (b), 40%-60% (c), 60%-80% (d), and 80%-100% data (e) as test sets. For the hold-out validation, we built the test sets by holding out data recorded from one institution at a time: VB (f), MD1 (g), MD2 (h), and CP (i).

stable conclusive results. Figure S17 displays the convergence of matrix A across different stages of patients. The Wasserstein distance is a metric for the differences between two distributions (the small distance value implies that two distributions are similar.) In Figure S17, we calculate the coupling matrices for different patients with different COPD stages by the length of the physiological signals (time-series) and calculate the Wasserstein distances between two neighboring time stamps (e.g., we calculate the Wasserstein distance between two coupling matrices generated from time-sequence 0 to t seconds and 0 to $t+1$ seconds). From Figure S17, we observed that—for each COPD stage—the Wasserstein distance of coupling matrices converges to a small value (less than 0.02) after 600 seconds. This observation suggests that after 10 minutes, the coupling matrices' elements remain constant. Thus, a 10 minutes record interval is sufficient for our model to make the predictions.

Early Viral infection detection

To emphasize the generality and correctness of our fractional dynamics method, we also analyze the Biochronicity viral prediction dataset with our model. The Biochronicity pilot study—relevant to the current work—is summarized as follows. The human rhinovirus (HRV)39 was injected into 18 human subjects monitored from 4 days before to 4 days after injection.

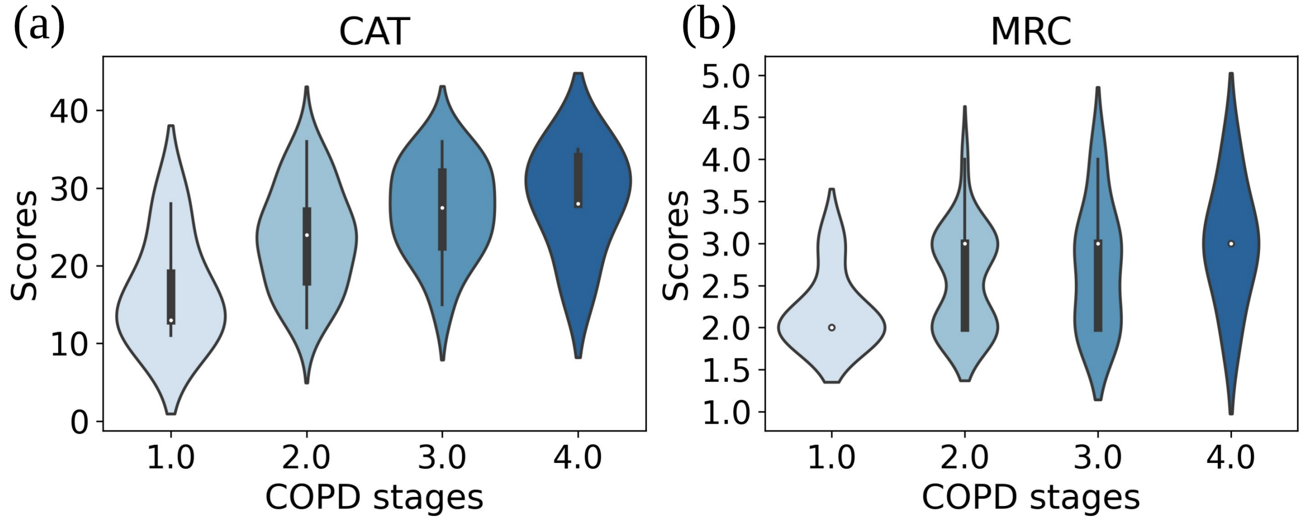

Figure S15. CAT (a) and MRC (b) scores for COPD patients across different COPD stages

Out of the 18 patients, 11 were shedding, showed symptoms after 3-4 days, and were marked as infected; the remaining 7 were considered healthy. The E4 Empatica physiological sensor records the temperature, blood pressure-volume (heart rate), accelerometer (3-axis), skin temperature, and electrodermal activity. The goal was to detect the viral infection in less than 24hrs from the inoculation point. The dataset imposes several practical challenges: first, paucity of data as there are only 18 samples; second, the detection has to be made as early as 24 hrs after the viral inoculation—when the symptoms are not prominent enough to be detected by a medical expert.

Next, we explain the fractional dynamics-based method/pipeline in viral prediction. We take the 3 physiological time-series features, electrodermal activity (EDA), body temperature (TEMP), and inter-beat interval (IBI). For a given inoculation point, the 3-dimensional time series are broken into pre- and post-viral infection data. For each pre- and post-viral data, a sliding window mechanism with a window length of 3000 samples and a sliding length of 100 samples (the choice is made by cross-validation over the window length and sliding length grid) is fitted using a fractional dynamical model with spatial coupling, from which we obtain the fractional coefficients α. Each window slide results in three fractional coefficients (one for each physiological feature), and then we estimate the probability density of α for pre- and post-viral periods. Finally, we use the Kullback-Leibler (KL) divergence between the pre- and post-viral fractional distributions as the feature for differentiating the infected and healthy subjects. The intuition is that the fractional coefficient captures the scaling behavior of the time series; by computing the difference between the distributions, we assume that healthy and infected subjects have different scaling behavior in their physiological activities.

One of the crucial assumptions in early viral detection is the knowledge of the viral inoculation point. From a practical standpoint, it is not possible to obtain such information. Therefore, we evaluate the efficacy of our model by moving the assumed viral inoculation point from the actual infection time in both directions. Figure S18 shows the plot of classification performance in terms of Type-I and Type-II errors when the inoculation point is shifted in positive and negative increments. Due to the scarcity of data, we use a leave-one-out-based resampling for the classification task. The 'V'-like structure of the Type-I error is informative because it shows the best performance (as few as 3 errors) close to the actual inoculation point (0 hrs) while the loss in performance on moving in either direction from 0 hours. The error increase (in either direction) is caused by shifting the inoculation point; the past and future of the patient become more or less the same. Hence, the classifier would falsely label the patient as healthy or, in other words, make a Type-I error. Another important observation is that the best performance—a total of 3 errors out of 18 subjects (3 Type-I and 0 Type-II)—is achieved not at the actual infection time (shift = 0 hours) but when the infection time is assumed delayed by +1 hour. A possible explanation is that the physiological effects take time to trigger; therefore, a delayed version of the separating boundary produces better differentiation between the pre- and post-viral data. Finally, another important outcome of the fractional model is that the Type-II error is roughly constant and is consistently low, which is required behavior.

WestRo Porti COPD dataset analysis

To test the generalization capabilities of our framework, we use the WestRo Porti COPD dataset consisting of 13824 physiological signals samples, recorded from 534 patients (232 COPD patients and 302 non-COPD patients) in the Victor Babes hospital.

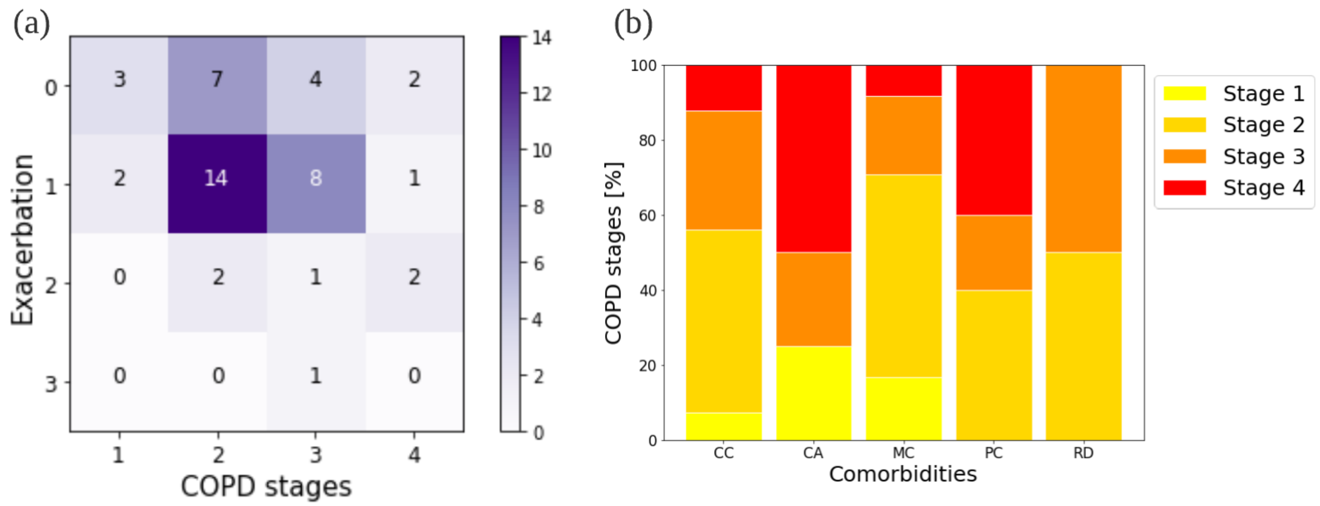

Figure S16. (a) Exacerbations across patients under different COPD stages (b) Percentage of COPD stages for each comorbidity (Cardiovascular comorbidities (CC), Cancers (CA), Metabolic comorbidities (MC), Psychiatric comorbidities (PC), Renal disease (RD)).

The dataset recorded 6 physiological signals from each patients (for detailed information about WestRo Porti COPD dataset, see the *Method* section *Data collection*). This section evaluates the accuracy, loss, and area under the curve (AUC). The results generated from different models (fractional-dynamics deep learning model, Vanilla DNN, LSTM, and CNN) are validated using the k-fold cross-validation approach (where $k = 5$). Figures S19 (a), (b), and (c) present our fractional-dynamics deep learning model's performance in terms of accuracy, loss, and AUC among training and testing datasets, respectively. Figure S19 (a) shows that both training and testing accuracy curves present an increasing trend—suggesting that our model improves the prediction accuracy over time without overfitting—to a predicting accuracy of $96.18\% \pm 0.48\%$. Figure S19 (b) illustrates that with increasing of the epoch numbers, the training and testing AUC curves also converge to optimal steady states. Figure S19 (c) shows that the loss curves for both training and testing processes exhibit a decreasing tendency, which indicates that, in our neural network, the value of the loss function converges over epochs.

Figure S19 (d), (e), and (f), respectively, show the training and testing accuracy, loss, and AUC curves obtained from the Vanilla DNN model trained with physiological signals (raw data). The testing results for the Vanilla DNN model show that the testing accuracy and AUC curves present a decreasing trend over epochs, which is evidence for overfitting. Consequently, we employ the early-stop mechanism to maintain the performance of the Vanilla DNN model (save the best-performance model). The best-performance model under Vanilla DNN exhibits a much lower prediction accuracy ($31.23\% \pm 4.58\%$) than the fractional dynamics deep learning model, which illustrates that our model outperforms the Vanilla DNN model.

Figure S19 (g), (h), and (i), respectively, present the training and testing accuracy, loss, and AUC results curves for the LSTM model trained with physiological signals (raw data). Observing the training and testing result curves in Figure S19 (g-i), we notice that the LSTM model also overfits; to deal with this situation, we also utilize the early-stopping mechanism to choose the best-performance LSTM model. The prediction accuracy of the best-performance LSTM model yielded $81.17\% \pm 2.52\%$, whereas our fractional dynamics deep learning model presents a significantly higher prediction accuracy of $96.18\% \pm 0.48\%$.

We also investigated whether the convolutional neural network (CNN) model can outperform our fractional-dynamics deep learning model by characterizing the dynamics of the physiological signals with higher accuracy. The results are presented in Figure S19 (j-l). Figure S19 (j), (h), and (i), respectively, show the training and testing accuracy, loss, and AUC results for the CNN model trained with physiological signals (raw data). The results show that the CNN model correctly classified $23.77\% \pm 0.02\%$ COPD samples under k-fold cross-validation ($k = 5$). Thus, our fractional dynamics deep learning model predicts patients' COPD stages with a much higher accuracy than the Vanilla DNN, LSTM, and CNN models—trained with physiological signals (raw data)—without overfitting (for detailed information about network architecture, see section *Methods*, subsection *Neural network architecture for the WestRo Porti COPD dataset*). Of note, the testing accuracy of $96.18\% \pm 0.48\%$ for the WestRo Porti COPD dataset is slightly lower than the testing accuracy for the WestRo COPD dataset. The reason is that the coupling matrix for the WestRo COPD dataset has more features than the coupling matrix for the WestRo Porti COPD dataset (i.e., 144 features vs. 36 features), and fewer features degrade the predicting accuracy.

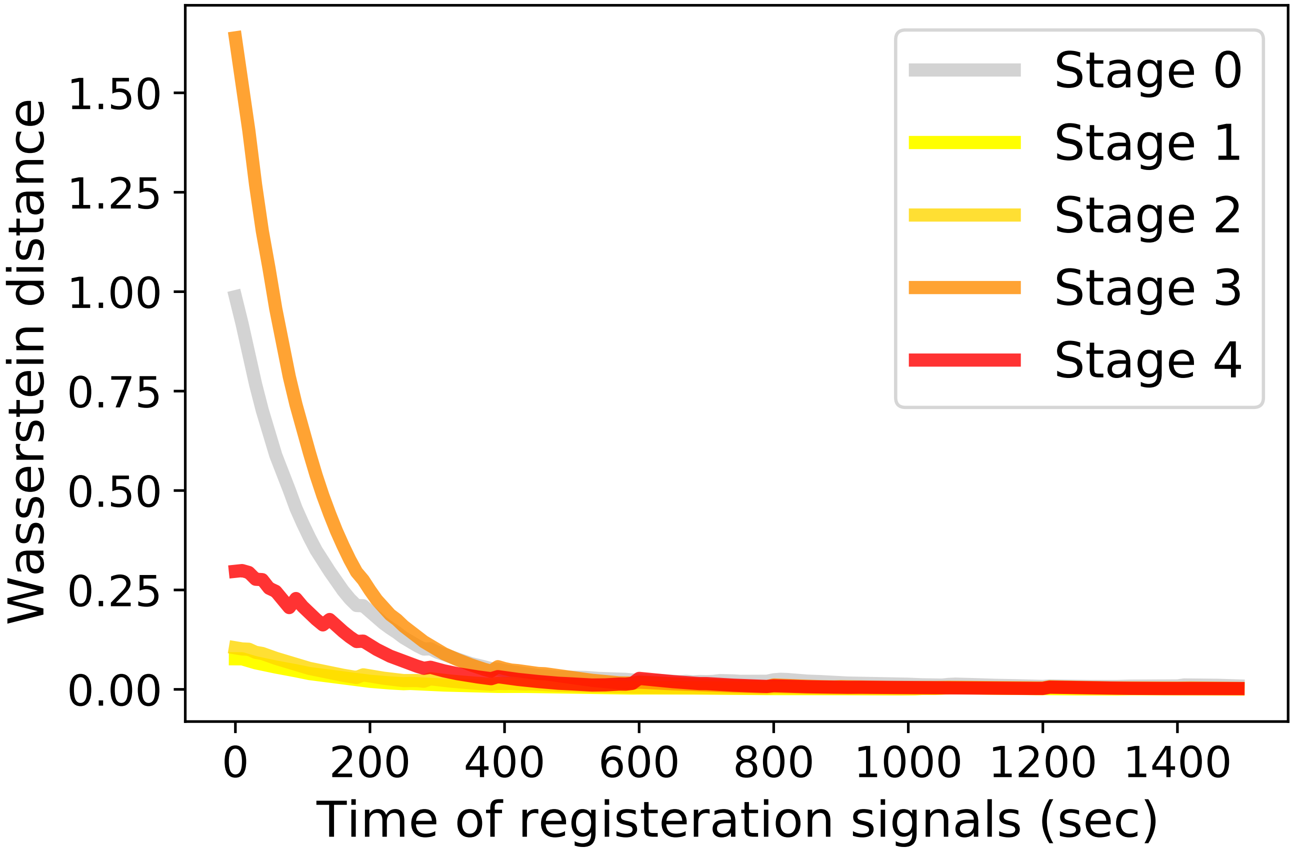

Figure S17. Wasserstein distance between the coupling matrices A over time.

Pre-patient based K-fold analysis

In this section, we performed the FDDLM's k-fold cross-validation results such that training does not use data from individuals considered in testing. (Nonetheless, our hold-out validation makes this type of evaluation implicit because each patient belongs to just one institution). Fig. S20 presents our model's performance as accuracy, loss, and AUC curves between training and testing datasets (where $k = 5$). Based on Fig. S20, we find that our model yielded an accuracy of $98.70\% \pm 0.407\%$ (very close to the accuracy we reported in the initially submitted manuscript).

References

1. Mukli, P., Nagy, Z. & Eke, A. Multifractal formalism by enforcing the universal behavior of scaling functions. *Phys. A: Stat. Mech. its Appl.* **417**, 150–167 (2015).
2. Hosmer Jr, D. W., Lemeshow, S. & Sturdivant, R. X. *Applied logistic regression*, vol. 398 (John Wiley & Sons, 2013).
3. Joachims, T. Training linear svms in linear time. In *Proceedings of the 12th ACM SIGKDD international conference on Knowledge discovery and data mining*, 217–226 (2006).
4. Bestall, J. *et al.* Usefulness of the medical research council (mrc) dyspnoea scale as a measure of disability in patients with chronic obstructive pulmonary disease. *Thorax* **54**, 581–586 (1999).
5. Jones, P. *et al.* Development and first validation of the copd assessment test. *Eur. Respir. J.* **34**, 648–654 (2009).
6. Halpin, D. M. *et al.* Global initiative for the diagnosis, management, and prevention of chronic obstructive lung disease. the 2020 gold science committee report on covid-19 and chronic obstructive pulmonary disease. *Am. journal respiratory critical care medicine* **203**, 24–36 (2021).
7. Wedzicha, J. A., Brill, S. E., Allinson, J. P. & Donaldson, G. C. Mechanisms and impact of the frequent exacerbator phenotype in chronic obstructive pulmonary disease. *BMC medicine* **11**, 1–10 (2013).

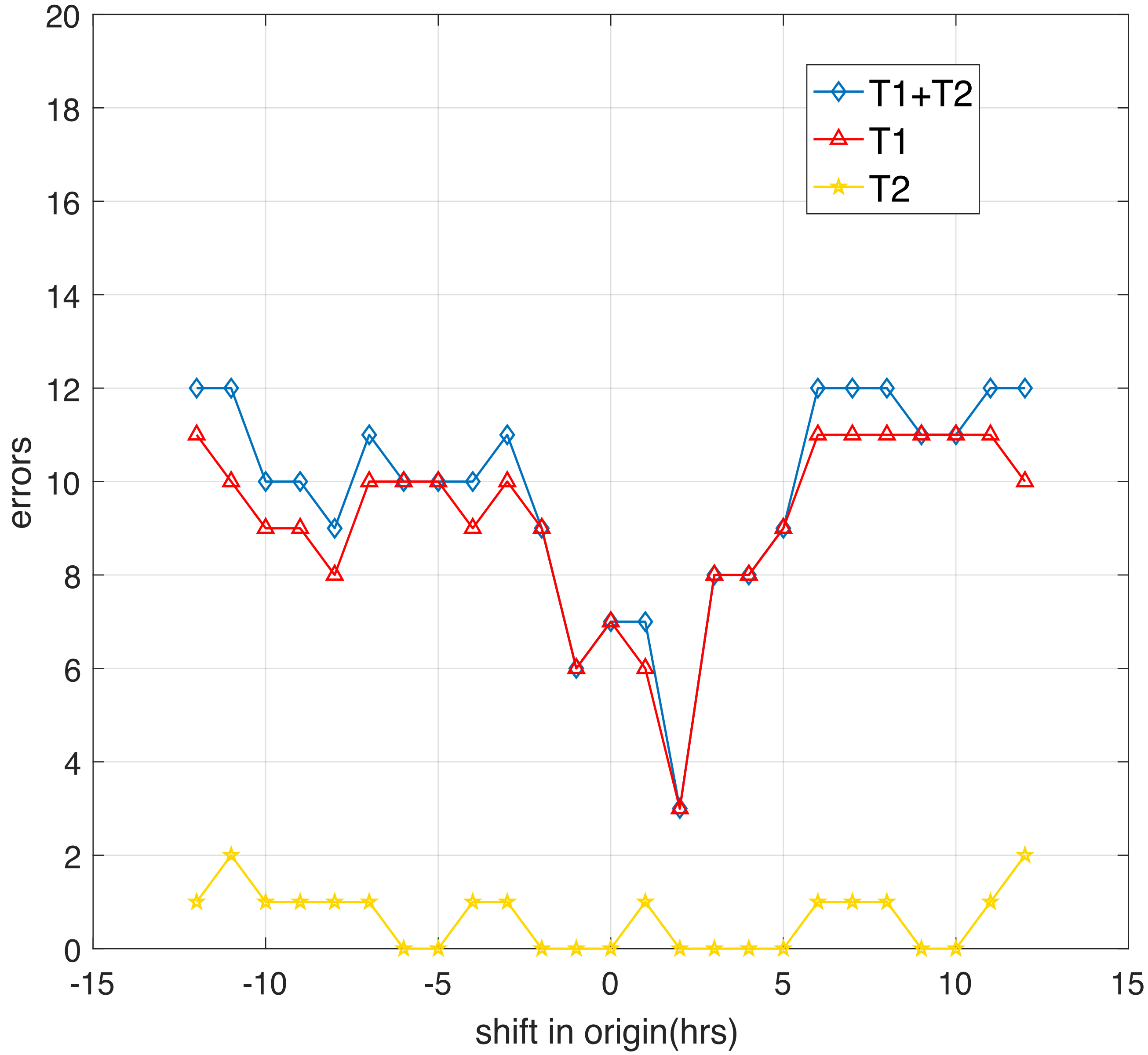

Figure S18. Early viral prediction using fractional dynamics. The Type-I and Type-II errors are shown by shifting the model's viral injection point from the actual reference (shift=0).

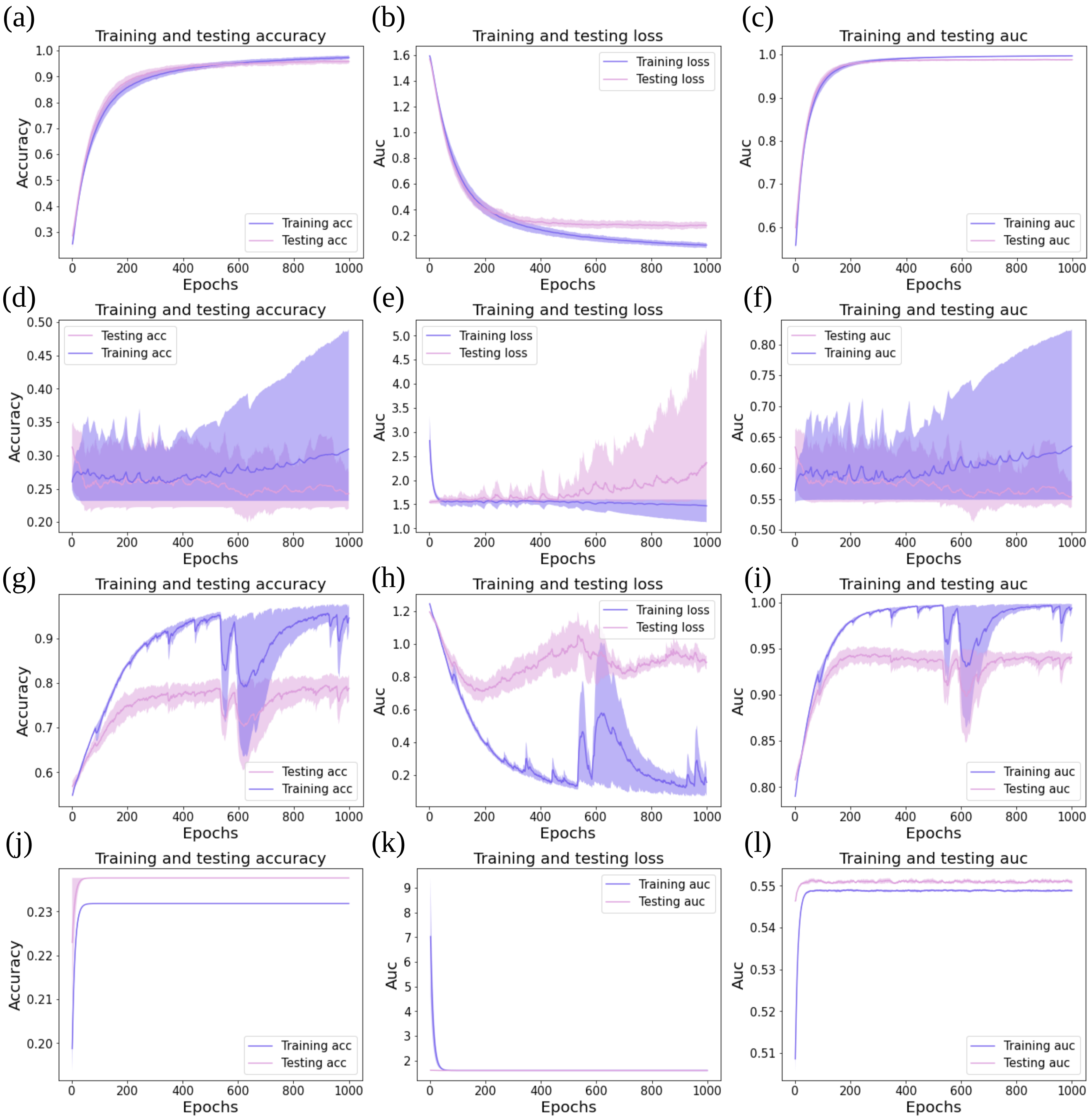

Figure S19. Training and testing result comparisons of different deep learning models (WestRo Porti dataset) for the *k*-fold cross-validation ($k = 5$). The training/testing accuracy (a), loss (b), and AUC (c) for our fractional dynamics deep learning approach, where the training processes use signal signatures extracted with the fractional dynamic mathematical model. The training/testing accuracy (d), loss (e), and AUC (f) for the Vanilla DNN model, where the training processes use the physiological signals recorded with the Porti portable sleep monitors. The training/testing accuracy (g), loss (h), AUC (f) for the LSTM model, where the training processes use the physiological signals. The training/testing accuracy (j), loss (k), and AUC (l) for the CNN model, where the models are trained with physiological signals.

8. Jo, Y. S. *et al.* Longitudinal change of fev1 and inspiratory capacity: clinical implication and relevance to exacerbation risk in patients with copd. *Int. journal chronic obstructive pulmonary disease* **14**, 361 (2019).

9. Halpin, D. M. *et al.* Effect of a single exacerbation on decline in lung function in copd. *Respir. medicine* **128**, 85–91

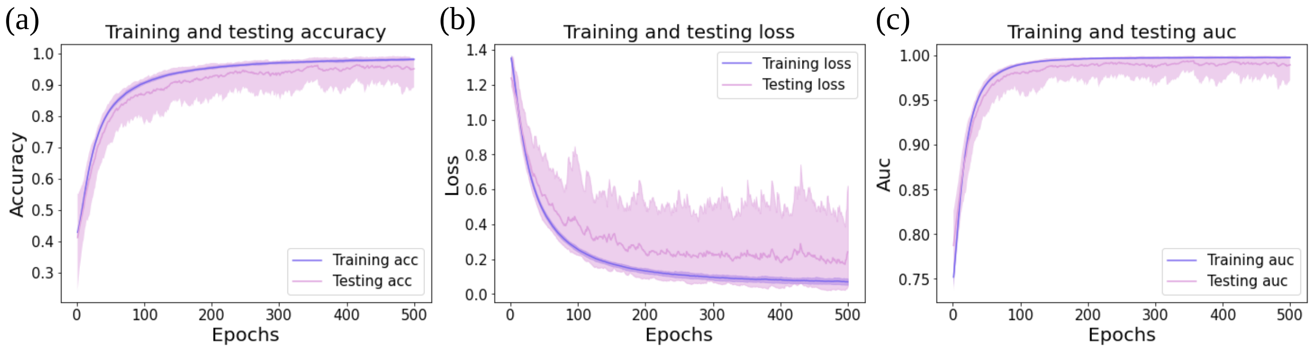

Figure S20. Training and testing result comparisons of different deep learning models (WestRo Porti dataset) for the *k*-fold cross-validation ($k = 5$). The training/testing accuracy (a), loss (b), and AUC (c) for our fractional dynamics deep learning approach, where the training processes use signal signatures extracted with the fractional dynamic mathematical model. The training/testing accuracy (d), loss (e), and AUC (f) for the Vanilla DNN model, where the training processes use the physiological signals recorded with the Porti portable sleep monitors. The training/testing accuracy (g), loss (h), AUC (f) for the LSTM model, where the training processes use the physiological signals. The training/testing accuracy (j), loss (k), and AUC (l) for the CNN model, where the models are trained with physiological signals.

(2017).

10. Agusti, A. *et al.* Characterisation of copd heterogeneity in the eclipse cohort. *Respir. research* **11**, 1–14 (2010).

11. Divo, M. *et al.* Comorbidities and risk of mortality in patients with chronic obstructive pulmonary disease. *Am. journal respiratory critical care medicine* **186**, 155–161 (2012).

12. Lopez-Campos, J. L., Ruiz-Duque, B., Carrasco-Hernandez, L. & Caballero-Eraso, C. Integrating comorbidities and phenotype-based medicine in patient-centered medicine in copd. *J. Clin. Medicine* **9**, 2745 (2020).
